# ASEAN countries’ environmental policies for the Sustainable Development Goals (SDGs)

**DOI:** 10.1007/s10668-022-02514-0

**Published:** 2022-07-23

**Authors:** Mark Elder, Gemma Ellis

**Affiliations:** Institute for Global Environmental Strategies, 2108-11 Kamiyamaguchi, Hayama, Kanagawa 240-0115 Japan

**Keywords:** Sustainable Development Goals (SDGs), ASEAN, Environmental policy, Voluntary National Reviews (VNRs)

## Abstract

This study’s objective is to examine how ASEAN countries reported their environment-related policies for the Sustainable Development Goals (SDGs) in their Voluntary National Reviews (VNRs), and a dataset of their environmental policies was developed. This is a necessary first step in analyzing the reasons for insufficient progress on the environmental dimension of the SDGs, since policies are key means of implementation. Previous studies of SDG progress and VNRs examined many aspects such as achievement levels, indicators, data, governance, and VNR preparation procedures, but surprisingly, there has been little discussion of countries’ actual policy efforts. Progress on the SDGs’ environmental dimensions is widely considered insufficient, including in Asia. This study showed that insufficient progress on the environmental dimension of the SDGs is not due to a lack of environmental policies or a lower prioritization of policies for environmental SDG targets. ASEAN countries included almost 600 concrete environment-related policies in their VNRs, widely distributed among most SDGs, accounting for about 40 percent of their total reported SDG-related policies. The number of environmental policies was not closely related to GDP, GDP per capita, or VNR date. Many policies appeared substantial, including national action plans, strategies, laws, and regulations, not just small projects or programs. However, some major existing environmental policies, for example on air pollution, were usually not included in the VNRs. Further research is needed to explore other possible factors such as insufficient policy implementation or effectiveness, which this study could not examine. This study’s environmental policy dataset provides the necessary baseline for future research on policy implementation and effectiveness, especially to help future studies identify ASEAN countries’ environmental policies in specific areas such as climate, energy, or water. This will also facilitate comparative analysis.

**Supplementary Information:**

The online version contains supplementary material available at 10.1007/s10668-022-02514-0.

## Introduction

The objective of this study is to examine how ASEAN countries reported their environment-related policy efforts for the Sustainable Development Goals (SDGs) in their self-reported Voluntary National Reviews (VNRs). This is a necessary first step in analyzing the reasons for insufficient progress on the environmental dimension of the SDGs, since policies are key means of implementation. One major contribution of this study is a dataset of the countries’ environmental policies, which could be the basis for further research on their effectiveness and implementation.

In the Anthropocene, the ecological and natural foundations of human society are endangered, and “planetary boundaries” are being crossed (Rockström et al., 2009; Steffen et al., 2007, 2015). Many now recognize that a “healthy planet” is necessary for “healthy people” (UNEP, 2019a). The Sustainable Development Goals (United Nations, 2015) encourage an integrated approach to strengthen human society’s ecological and natural foundations while promoting human well-being (Biermann et al., 2017; Chasek & Wagner, 2016; Elder & Olsen, 2019; Griggs et al., 2013).

However, it is widely recognized that there has been insufficient progress on the SDGs, especially their environmental dimensions, even before COVID-19 (Independent Group of Scientists, 2019; Sachs et al., 2020; United Nations, 2020), including in the Asia–Pacific (UNESCAP, 2019, 2020, 2021, 2022). Only 22 of 93 environment-related SDG indicators made good progress over the previous 15 years, and there are insufficient data to assess progress on 68 percent (UNEP, 2019b). UNESCAP concluded that the Asia–Pacific region is “going backwards” on SDGs 12 (responsible consumption and production) and 13 (climate). One study noted that progress on the major environment-related SDGs 12, 14, and 15 in the Asia–Pacific has been “limited” (ADB, 2019).

Policies are key means for national governments to implement SDGs. Therefore, it is important to analyze them in order to understand the reasons for insufficient progress.

However, most existing assessments and studies of SDG progress and VNRs have not addressed actual efforts—especially policies (Elder & Bartalini, 2019). Instead, existing studies have addressed many other aspects including achievement levels and the need for better indicators and data (Allen et al., 2017, 2020; Guppy et al., 2019; Sachs et al., 2020; Sciarra et al., 2021; UNEP, 2019b; United Nations, 2020, 2021); governance issues such as establishing SDG coordination frameworks, mainstreaming SDGs in national policies/frameworks and budgets, and multistakeholder participation in SDG processes, etc. (Okitasari et al., 2019; Sunam et al., 2018); the need to strengthen monitoring and evaluation (IIED et al., 2017; Yonehara et al., 2017); and interlinkages (including synergies and trade-offs) among SDGs (Alcamo et al., 2020; Allen et al., 2018; Breu et al., 2020; Elder et al., 2016; Elder & Olsen, 2019; Kroll et al., 2019; Nilsson et al., 2016; Weitz et al., 2018; Zhou & Moinuddin, 2017). One study used content analysis to determine how the VNRs of 20 European countries addressed health issues, but it did not directly examine to what extent the VNRs mentioned concrete policies (Bickler et al., 2020). The UN’s 2020 and 2021 VNR Synthesis Reports briefly discussed national implementation, noting many countries’ progress linking the SDGs with national development plans, mapping national budgets with SDGs, and establishing national SDG coordination procedures (UN DESA, 2020, 2021). The reports’ surveys of individual SDGs mentioned some examples of implementation policies in addition to discussions of goal attainment. However, the reports did not systematically compile or analyze concrete implementation policies.

Consequently, the *Sustainable Development Report* 2020 identified a need to assess SDG policy implementation (Sachs et al., 2020). This study aims to respond to this need. Identification of countries’ SDG-related policies is a necessary but very difficult first step to assess their implementation.

The *Sustainable Development Report 2021* (with SDG Index and Dashboards) (Sachs et al., 2021) proposed a method to assess SDG policy implementation which uses quantitative indices as a proxy for policies. This facilitates comparison between countries. Their report proposed four environmental policy indicators under the heading “energy decarbonization and sustainable industry”: (1) UN Climate Ambition Alliance signatory, (2) policy or NDC-based commitment to reach net zero emissions by 2050, (3) 1.5-degree Celsius Paris Agreement-compatible climate action (Climate Action Tracker), and (4) unconditional fossil fuel subsidies (Energy Policy Tracker), which were presented for G20 countries. Their approach can be useful when quantitative data or existing policy trackers are available, but this is not the case for many environment-related targets. Four indicators are not sufficient to analyze countries’ policy efforts on the full range of environmental targets. Moreover, detailed information on specific policies is needed to construct policy indices or trackers as well as to analyze the effectiveness of specific countries’ policies in detail; simply using an index would not be sufficient.

Only two studies have produced datasets of countries’ actual concrete SDG-related policies based on VNRs, one on the G20 countries (Elder & Bartalini, 2019) and one on the ASEAN countries (Elder, 2020). The authors are aware of only one other study on the environmental dimensions of the SDGs in ASEAN countries which includes a discussion of actual policies. This report by ADB examined the environmental dimensions of SDGs in the Asia–Pacific, but it was based on a workshop and interviews of policymakers, not a systematic study of VNRs or actual policies, and it mainly focused on only 3 SDGs (12, 14, and 15) plus a few other targets. It did not address all environment-related targets (ADB, 2019). Other studies focused on topics like health (Bickler et al., 2020) or specific SDGs. For example, SDG 8 is widely understood to be on economic growth and decent work, but it also includes a key environmental target (8.4) on decoupling “economic growth from environmental degradation” (ILO, 2019; UN DESA, 2018). The IMF published a paper on its role in facilitating ASEAN’s progress on the SDGs; it provides a general overview but discussion of the environment is limited, and it does not address ASEAN’s environmental policies (IMF, 2018).

This study examines ASEAN countries’ environment-related SDG efforts, focusing on policies, and it develops a dataset of the environment-related policies listed in their VNRs, based on a previous study which comprehensively identified the SDG policies of the ASEAN countries contained in their VNRs (Elder, 2020). The number of policies listed by each country can be considered a very rough indicator of effort, as well as the policies’ distribution among the SDGs, which may indicate some prioritization.

Five hypotheses about the relationship between the environmental policies listed in the countries’ VNRs and insufficient progress on the environmental dimensions of the SDGs are examined by this study. The natural first hypothesis is that countries might have adopted few environment-related SDG policies. Doubts about countries’ policy efforts are understandable, and it is not easy to obtain information on them, especially on 169 targets. Countries’ presentations at the High-Level Political Forum (HLPF) are extremely short, so there is little time to present policies (Beisheim, 2020), much less explain them in detail. The VNRs themselves contain some detailed information, but they are very long and require considerable effort to read. SDG-related policies not mentioned in VNRs are even more difficult to observe. Reading the VNRs themselves is the only practical way to obtain substantial information about implementation policies on a wide range of SDGs in a reasonable time period.

Certainly, the number of policies by itself is not the best indicator of effort or prioritization since the policies are difficult to compare using clear criteria. The significance of the individual policies ranges widely. Some are broad strategies incorporating many specific policies in different areas, while others are small projects. Many do not have easily observable effective dates. Moreover, this study cannot assess the degree of policies’ implementation or effectiveness, which should be a major focus of future SDG research. Nevertheless, the compiled database of policies is valuable, and it is necessary to count the number of policies included. The presence or absence of policies provides a basic initial indication of effort.

The second hypothesis is that countries might have a lower share of environment-related policies compared to non-environmental ones, or they might prioritize SDGs and SDG targets which are less environment-related. Third, policies listed in the VNRs might be mainly small-scale projects or initiatives rather than major, substantial policies. Fourth, developing countries with lower per capita incomes might have fewer policies in general, and/or a smaller share of environment-related policies. Fifth, larger countries with higher levels of GDP—even larger developing countries—might have more environmental policies in general and a higher share of environment-related policies than smaller countries.

The results did not support any of these hypotheses. The ASEAN countries listed a significant number of environment-related policies in their VNRs, which were widely distributed among a range of SDGs. Almost 600 policies were listed by the 9 countries submitting VNRs, accounting for almost 40 percent of the total number of their policies. Many policies were quite substantial, including national action plans, strategies, and regulations, not just small projects or programs. Moreover, the VNRs appear to under-report some major existing environmental policies. Therefore, insufficient progress on the environmental SDGs in ASEAN countries cannot be attributed to a lack of policies in general, or a lack of environment-related policies, and further research is needed to explore other factors such as insufficient policy implementation or effectiveness. The results also showed that the number and distribution of policies in the VNRs were not closely related to GDP, GDP per capita, or date of VNR.

Developing the dataset of environment-related policies is a necessary first step in analyzing their effectiveness, and it required a major research effort. The environment is a very complex policy area, and most countries have many policies. Information on policies is often not easily available in English. Therefore, the SDG VNRs provide a rare opportunity to obtain information on policies, in English, on many policy areas (not only the environment). It was probably not easy for countries themselves to compile this information, which may partly explain the VNRs’ apparent incompleteness. Normally, a well-funded research effort involving local consultants is necessary to obtain even basic information in English about policies in one country on one specific topic, especially since this information is often available only in local languages. Fortunately, all of the ASEAN countries’ VNRs were submitted in English and include information about policies on a wide range of issues. Since VNRs are official government documents, the information contained in them, including English translations of policy names and brief English explanations, is in principle recognized by the government submitting the VNR. Thus, the information in the VNRs facilitates more detailed research within individual countries as well as comparative policy research.

In the rest of this paper, Sect. 2 discusses the background and context, and Sect. 3 explains the methodology. Section 4 presents the comparative analysis, Sect. 5 is the discussion, and Sect. 6 concludes. The Electronic Supplementary Material contains additional detailed discussion of individual countries (ESM 1) and the environmental policy dataset (ESM 2).

## Background and context

For the past several decades, ASEAN countries have experienced remarkable, rapid economic growth and poverty reduction which was accompanied by serious environmental problems including severe air and water pollution, deforestation, and greenhouse gas emissions (GHGs) (Kojima & Bengtsson, 2015). The ASEAN countries have made various efforts and adopted a variety of policies to address these problems over the years with varying degrees of effectiveness. Still, many of these environmental problems remain serious or are worsening, such as GHG emissions and marine pollution (not only in ASEAN, but also in other countries).

Much past research on the environmental policies of ASEAN countries has generally focused on specific environmental policy issues, either in single countries or cities, or in a comparative context (often including non-ASEAN countries), such as circular economy (Akenji et al., 2019), water (Ministry of the Environment of Japan and IGES 2019), air pollution (Elder, 2015), low carbon transport (Zusman et al., 2012), marine plastic litter (Hermawan & Astuti, 2021), fuel quality and vehicle emission standards (UNEP, 2014), recycling (Drafting Committee, 2018), etc. Some studies focused on law and governance (Elliott, 2012; Koh, 2013; Mori, 2013). There have also been studies of regional cooperation efforts (Elliott, 2017) to address these problems. Some of these efforts, such as the ASEAN Haze Agreement, are within ASEAN itself (Lee et al., 2016; Muhammad, 2021; Nurhidayah et al., 2014; Varkkey, 2014). ASEAN has published five State of the Environment Reports which mention some examples of countries’ policies, but they do not analyze the policies comprehensively or systematically (ASEAN, 2009, 2017). These reports also discuss member countries’ engagement with a range of Multilateral Environmental Agreements. There are many other environmental cooperation frameworks outside of ASEAN which include ASEAN countries (Elder, 2018).

It is hoped that the SDGs will further encourage the ASEAN countries’ environment-related efforts. ASEAN countries have tended to prioritize the economic dimensions of development, and one of the strengths of the SDGs is their potential to encourage more attention to the environmental dimension by demonstrating potential synergies with the economic and social dimensions. Certainly, the ASEAN countries have enthusiastically engaged with the SDG process, and they worked hard to produce substantial VNRs. Moreover, the VNRs provide a unique opportunity to generate a broad-ranging dataset of the countries’ environmental policies to facilitate further analysis.

## Methodology

This study uses a broad concept of the environmental dimensions of the SDGs based on a framework (Elder & Olsen, 2019) which is similar to UNEP’s (UNEP, 2019b). The framework’s key point is that important environment-related targets are included in almost all SDGs, not just SDGs 12–15, which are sometimes considered the “environmental” SDGs. Key environmental targets outside of SDGs 12–15 include 2.4 (sustainable food production), 3.9 (pollution-related deaths and illness), 4.7 (education for sustainable development), 6.5 (water ecosystems), 7.2 (renewable energy), 7.3 (energy efficiency), 8.4 (resource efficiency and decoupling economic growth from environmental degradation), 9.4 (sustainability upgrading of industry and resource efficiency), etc. The environment-related targets outside of SDGs 12–15 are listed in Elder and Olsen (2019).

Identifying the environment-related policies involved four steps. First, the basis was a comprehensive list of all SDG policies reported in the ASEAN countries’ VNRs as compiled in a previous study (Elder, 2020). Second, the environment-related policies were identified from this compilation using a broad interpretation based on key words such as “environment,” “sustainability,” “pollution,” or whether the policy name was related to environmental issues. Third, in cases where it was difficult to determine whether the policy was environment-related, the text of the original VNR, or the policy itself (if available in English) was checked, especially for large-scale and broad-ranging national policies and strategies. When a policy was listed under more than one SDG, only the environment-related listings were included. Fourth, the list of policies was compared with the list of environmental targets again to check whether some policies could be considered under more than one SDG. Repeated policies were counted according to the number of SDGs that they could be listed under. The total number of policies with and without counting the repeated policies is reported separately. The dataset of each country’s environment related policies arranged by SDG is in the Electronic Supplementary Material (ESM 2).

It is important to understand some differences in the overall context of the countries’ VNRs. Nine of the ten ASEAN countries had published at least one VNR at the time the research for this study was conducted. Only Myanmar had not published one. Indonesia and the Philippines published two. The Philippines’ first VNR was excluded from this analysis since it did not list any policies, probably because it was one of the first countries to produce a VNR in 2016 when there were no previous examples to follow. Moreover, Indonesia and the Philippines only addressed some, not all, of the SDGs in their VNRs, mainly the SDGs that were highlighted at the HLPF during the year that the VNRs were produced. The policies in Indonesia’s two VNRs were combined in this analysis. Explaining why different countries prepared their VNRs with different formats using different methods is beyond the scope of this study and would require interviews with the relevant policymakers since the relevant information is not included in the VNRs themselves.

## Results: comparative analysis

### Number of environment-related policies and their share in the total number of policies

Table 1 calculates the total number of each country’s environment-related policies. Column A lists the total number of each country’s policies for all SDGs. Column B adds the policies repeated by countries in their VNR (under more than one SDG). The total number of policies directly listed in the VNRs by the countries, including repeated ones (combining columns A and B), are in column C. Column D reports additional repeatable policies which appear to address additional SDGs, but which were not directly repeated by the countries in their VNRs. Column E totals all these environmental policies. Column A is the most conservative count, column E is the most generous count, and column C is between them.

**Table 1:** Environment-related policies by country

Country	Total number of SDG policies^a^	Total Individual env. policies in VNRs (original)		Env. policies repeated in VNRs (original)		Total env. policies reported by countries (original)		Additional repeatable env. policies (adjusted)		Total adjusted env. policies	
		Number	% of total SDG policies	Number	% of total env. policies reported by countries	Number	% of total SDG policies	Number	% of total adjusted env. policies	Number	% of total SDG policies
Column =>	T	A		B		C		D		E	
Calculation =>			A/T		B/C	A + B	C/T		D/E	C + D	E/T
Brunei Darussalam	270	**59**	21.9	6	9.2	**65**	24.1	16	24.6	**81**	30.0
Cambodia	128	**42**	32.8	9	17.6	**51**	39.8	23	45.1	**74**	57.8
Indonesia	225	**46**	20.4	2	4.2	**48**	21.3	28	58.3	**76**	33.8
Lao PDR	77	**29**	37.7	6	17.1	**35**	45.5	10	28.6	**45**	58.4
Malaysia	130	**56**	43.1	10	15.2	**66**	50.8	21	31.8	**87**	66.9
Philippines	92	**30**	32.6	3	9.1	**33**	35.9	14	42.4	**47**	51.1
Singapore	235	**101**	43.0	4	3.8	**105**	44.7	37	35.2	**142**	60.4
Thailand	215	**89**	41.4	0	0	**89**	41.4	12	13.5	**101**	47.0
Viet Nam	201	**95**	47.3	6	5.9	**101**	50.2	22	21.8	**123**	61.2
Total	1,573	**547**	34.8	46	7.8	**593**	37.7	183	23.6	**776**	49.3
Average	174.8	**60.8**	35.6	5.1	9.1	**65.9**	39.3	20.3	33.5	**86.2**	51.8

*Source* Compiled by authors

^a^Total number of SDG policies (all SDGs): (Elder, 2020)

Even the conservative count clearly shows that each ASEAN country listed many environment-related policies in their VNRs, ranging from 21 (Lao PDR) to 101 (Singapore), 547 in total, averaging over 60 per country. The count in column B is similar, ranging from 33 to 105 (Philippines and Singapore, respectively), almost 600 in total, and averaging almost 66 per country. The most generous count ranges from 45 to 142 policies (Lao PDR and Singapore), 776 policies in total, averaging over 86 per country.

Perhaps surprisingly, the total number of environment-related policies is a significant share of all the SDG policies in the VNRs. Even the conservative number averaged over one-third of the total number of policies, ranging from a low of 20.4 percent (Indonesia) to a high of 47.3 percent (Viet Nam). Column B is the most appropriate comparison, since it uses the same counting method that was used to calculate the total number of SDG policies (Elder, 2020). This method shows a slightly higher share of environment-related policies, ranging from a low of 24.1 percent to a high of 50.2 percent (Indonesia and Vietnam, respectively). Column C somewhat overstates the share of environment-related policies. The number for Indonesia, however, is clearly understated, since Indonesia did not review some SDGs in its two VNRs, even though it clearly has environment-related policies in these areas. The number for the Philippines is rather high (almost 36 percent) considering that its VNR only covered the SDGs highlighted at the 2019 HLPF, and so it did not include policies for major environment-related SDGs such as 6, 7, 12, 14, and 15. In contrast, Brunei Darussalam’s VNR was lower than expected since its VNR was the most recent (2020), covered all SDG, and had the largest total number of policies, but it had the second lowest share of environment-related policies.

In interpreting these results, columns A and C represent the understanding of each country’s government, at least the divisions which prepared and approved the VNR. However, clearly, countries did not fully recognize the extent to which some policies may apply to other SDGs, so column E better represents the actual environmental scope of the policies listed in the VNRs.

Repetition of policies indicates that countries recognized that some policies apply to more than one SDG. All but one country repeated at least a few policies, ranging from 15 to 18 percent in the three countries with the highest share of repeated policies. However, countries did not recognize this systematically, and further analysis showed that many other policies listed by the countries could apply to more than one SDG, ranging from 13.5 percent of Thailand’s environment related policies to over 58 percent of Indonesia’s policies.

In particular, most climate policies are related to SDGs other than 13, and several countries did not list their SDG 13 policies under other related SDGs. For example, Brunei Darussalam listed four policies related to energy efficiency under SDG 13, but not under SDG 7, even though these are also directly related to SDG 7.3 (energy conservation). Cambodia listed four policies on water quality and wastewater treatment under SDG 11 (on cities) but not under SDG 6 (on water) even though they are directly related to SDG 6.3. Policies related to sustainable consumption and production under SDG 12 are also generally related to SDGs 8 (especially target 8.4 on decoupling and resource efficiency), 9 (sustainable industrialization), or 7 (targets 7.2 on renewable energy and 7.3 on energy efficiency), but they are not always listed under all related SDGs.

### Distribution of policies among SDGs

Table 2 indicates the distribution of environment-related policies among SDGs. The results from columns C and E in Table 1 are reported separately.

**Table 2:** Environment-related policies by SDG

Country	No. of policies directly related to the environment under each SDG																											
	1c	1d	2c	2d	3c	3d	4c	4d	5c	6c	6d	7c	7d	8c	8d	9c	9	10c	11c	12c	13c	14c	15c	16c	17c	Total policies (c)	Total repeatable policies (d)	Sum total (e)
Brunei Darussalam	1	3				1		1	1	3	3	3	5	3		1	3		13	6	10	9	15			**65**	16	**81**
Cambodia	1	1	1	1	1	3	1	2	2	5	12	1	2	1	2	1			10	7	8	8	4			**51**	23	**74**
Indonesia	2	8	2	3			1				7		5	2	1	2	4			1	22	9			7	**48**	28	**76**
Lao PDR			2	1						3	4	1	1		2	1	2		5	5	8		10			**35**	10	**45**
Malaysia		1	2	1		1		2		1	7	4	2	1	2	6	5		6	8	5	17	16			**66**	21	**87**
Philippines		7		1							2		3	7			1				26					**33**	14	**47**
Singapore			3	1		5		1		11	13	5	9			1	8		13	33	19	8	10		2	**105**	37	**142**
Thailand	3	1	5	1			2			4	3	5	3	3	1	9	3		4	9	7	22	15		1	**89**	12	**101**
Viet Nam		4	3	1		1				11	7	4	4	6	1	3	4		8	16	20	14	16			**101**	22	**123**
Total policies (c)	**7**		**18**		**1**		**4**		**3**	**38**		**23**		**23**		**24**		**0**	**59**	**85**	**125**	**87**	**86**	**0**	**10**	**593**		
Total repeatable policies (d)		25		10		11		6			58		34		9		30										183	
**Sum Total (e)**		**32**		**28**		**12**		**10**	**3**		**96**		**57**		**32**		**54**	**0**	**59**	**85**	**125**	**87**	**86**	**0**	**10**			**776**

c = The SDG under which the policy was placed in the VNR. This is from column (C) in Table 1

d = The SDG which the policy also addresses (repeatable policy). This is from column (D) in Table 1

e = This is c+d, from column (E) in Table 1

*Source* Compiled by authors

ASEAN countries listed the most policies under SDG 13, followed closely by 14, 15, and 11. Nevertheless, many policies were also listed under other SDGs which are not always considered as “environmental,” especially SDGs 2, 6, 7, 8, and 9. The smallest number of policies was listed under SDGs 3 and 4.

The percentage of environment-related SDGs outside of SDGs 12–15 ranges from 30 to 45 percent. However, the Philippines is not included in this because its VNR did not report on all SDGs (only the SDGs highlighted at the HLPF in the year it issued the VNR), so it does not mean that it does not have policies relating to the non-reported SDGs.

“Repeatable” environmental policies, not indicated directly by the countries in their VNRs, were found under all SDGs from 1–9 (except 5), especially in SDGs 1, 6, 7, and 9.

Table 3 discusses observations on specific SDGs.

**Table 3:** Observations on specific SDGs

SDG	Observations
1	• This focuses on poverty so its relation to the environment is relatively indirect • Target 1.5 is on climate resilience, so policies should generally relate to SDG 13
2	• Agriculture is a priority for most countries, and interest in sustainable agriculture is increasing, so the number of policies seems low • Some climate policies listed under SDG 13 were related to sustainable agriculture
3	• Several countries mentioned policies related to chemical pollution and hazardous materials under SDG 12, but this is also related to SDG 3 • SDG 3 refers to pollution (air, water, and land), and countries do have policies in these areas. It is not clear why these policies were mostly not mentioned in VNRs
4	• The VNRs often linked education for sustainable development with climate change
5	• Gender is linked indirectly to the environment in the SDGs, relating to women’s ownership of land and natural resources. However, generally, there are strong links (UNEP, 2016, 2019a) • Only Cambodia linked gender and environment
6	• Water is a major environmental issue, and all countries except the Philippines and Indonesia listed policies. (This is because SDG 6 was not highlighted at the HLPF in the years when Indonesia and the Philippines produced their VNRs. Certainly, these countries have water-related policies.) • Many other policies relating to SDG 6 can be found throughout the countries’ VNRs, especially under SDGs 11, 12, and 14
7	• Countries listed many energy-related policies under SDGs 12 or 13 instead of 7
8	• Most countries mentioned policies related to sustainable tourism, though many were listed under other SDGs • Policies related to resource efficiency are also related to SDGs 9 and 12 • Most countries did not address “decoupling,” though they may have related policies • This study considered policies on green growth or similar concepts to be related to SDG 8, although these policies were usually listed under other SDGs
9	• All countries have policies related to SDG 9 which are listed under other SDGs, especially on transport (generally listed under SDG 11)
11	• Many policies under SDG 11 are also related to SDG 9 • Cambodia and Singapore have the most policies. Cambodia’s policies focused mainly on waste, while Singapore’s focused on public transport
12	• This SDG has the third highest number of policies • Only the Philippines did not list any policies, although it definitely has some (Ipac, 2019)
13	• SDG 13 has the most environment related policies compared to others • Every country listed policies under SDG 13
14	• All countries, except Lao PDR (the only landlocked ASEAN country) and the Philippines listed at least eight policies
15	• Only Indonesia and the Philippines did not address this SDG, since it was not highlighted at the HLPF in the years that the countries issued their VNRs • Cambodia listed only four policies, but others listed between 10 and 16
16	• No environment-related policies were listed here
17	• SDG 17 does not have any targets directly related to the environment, but three countries listed environment-related policies here, mainly partnerships to implement various initiatives or innovative financing, such as green bonds

ASEAN countries generally recognized the environmental elements of the SDGs which are sometimes considered “non-environmental” and listed environmental policies under them. However, some countries seem to have overlooked some cases, which this study identified as “repeatable” policies, and generally, their distribution was significantly different from the policies listed directly in the VNRs. The major exception was SDG 1; the countries actually mentioned some related environmental policies, but they were listed under other SDGs instead. Cambodia listed at least one environmental policy under most SDGs except 10, 16, and 17. It was also the only country to list a policy under SDG 3.

### Under-reporting of policies

Overall, the number of environment-related policies seems to be under-reported in the VNRs, despite countries’ inclusion of them across a range of SDGs. There are three main types of cases of this. First, for several environment-related SDG targets, many countries listed few or no policies even though most countries typically have related policies. The most notable one is probably SDG 3, since it includes targets on deaths and illness related to all forms of pollution as well as from hazardous chemicals. Few countries reported air pollution policies here, or under other SDGs with air pollution related targets—11.6 on reducing the environmental impact of cities, “paying special attention to air quality” and 12.4 on achieving “environmentally sound management of chemicals and all wastes” and significantly reducing their “release into the air…” (Elder & Zusman, 2016)—﻿even though countries clearly have policies on air pollution, chemicals, waste, etc. (Akenji et al., 2011; Drafting Committee, 2018; Elder, 2015; Ministry of the Environment of Japan and IGES 2018).

Second, some countries (e.g., Cambodia) listed many large scale, long-term and/or national policies which can apply to many SDGs (for example, the National Strategic Development Plan), but often these were only listed under one SDG (Kingdom of Cambodia, 2019). Third, two countries did not report on all SDGs, focusing only on the ones highlighted at the HLPF in the year the VNR was produced. Thus, even though Indonesia issued two VNRs, they did not systematically cover the SDGs which were not highlighted during those two years (despite a few exceptions) (Republic of Indonesia, 2017, 2019). The Philippines did not highlight any specific policies in its first VNR, which was one of the earliest (2016), and in its 2019 VNR, it only reviewed the SDGs highlighted at that year’s HLPF (Philippines, 2016, 2019). Nevertheless, clearly Indonesia and the Philippines do have policies addressing the other SDGs, but they simply were not reported in the VNRs.

### Relation to GDP, GDP per capita, and VNR year

This section examines whether the number of environmental policies reported in the VNRs was related to GDP, GDP per capita, or the year the VNR was prepared. Table 4 shows that environmental policies do not appear to be very correlated with GDP per capita, total GDP, or the year the VNR was prepared, regardless of the counting method. To some extent, countries with higher/lower GDP per capita were associated with a higher/lower number of policies, but there were some notable exceptions. Vietnam, with the second lowest per capita income, had the second highest number of policies. The country with the highest percent of environment related policies, Malaysia, ranks third (out of 9), while Viet Nam, with the second highest percent of environment related policies, has the second lowest per capita GDP. Previous studies found some degree of correlation between the total number of policies and the year of the VNR (Elder, 2020; Elder & Bartalini, 2019), but the correlation does not seem to apply to environment related policies. The Philippines was one of the first countries to submit a VNR in 2016, and its VNR did not list any specific policies, which is understandable. However, its 2019 VNR lists about the same amount or fewer environmental policies compared to other ASEAN countries that published VNRs in the same year or earlier. Thailand was the first country to report specific environmental policies in 2017, which remained the third highest amount even in 2020. In contrast, Brunei Darussalam was the latest ASEAN country to issue a VNR, but its total number of environment related policies was about the same as the region’s average.

**Table 4:** Number of environment-related policies compared with total and per capita GDP and VNR year

Country	GDP Per capita (2018) (World Bank)	Total GDP (2018) (World Bank)	Latest VNR Year	Env. policies reported by countries (C)^a^		Total adjusted env policies (E)^a^	
	USD	USD (mil.)		Number	Percent	Number	Percent
Singapore	64,582	364,157	2018	105	44.7	142	60.4
Brunei	31,628	13,567	2020	65	24.1	81	30.0
Malaysia	11,239	354,348	2018	66	50.8	87	66.9
Thailand	7,274	504,993	2017	89	41.4	101	47.0
Indonesia	3,894	1,042,173	2019	48	21.3	76	33.8
Philippines	3,103	330,910	2019	33	35.9	47	51.1
Lao PDR	2,568	18,131	2018	35	45.5	45	58.4
Vietnam	2,564	244,948	2018	101	50.2	123	61.2
Cambodia	1,512	24,572	2019	51	39.8	74	57.8
Average	321,978	14,263	2018	66	39.0	86	52.0
Total	2,897,799			593		776	

^a^From Table 1

A more detailed discussion of individual countries is included in the Electronic Supplementary Materials (ESM 1).

### Types of policies

It is important to note that in all ASEAN countries, many of the policies listed appeared to be substantial (see ESM 2.1–2.9). Various national action plans and strategies were most notable. Many could be voluntary, but they often have many components covering a variety of areas. Nevertheless, countries also listed many laws and regulations as well as specific infrastructure projects. Less common substantive policies included tax/spending measures, protected areas/parks, pricing, enforcement, monitoring, certification/labelling systems, green procurement, and research and data collection. Most policies were not awareness-raising campaigns, training/capacity building, telephone hotlines, or small projects, although these were also included.

## Discussion

The results did not support the five hypotheses. Regarding the first hypothesis, the results showed that insufficient progress on the environmental dimension of the SDGs in ASEAN cannot be attributed to a simple lack of environment-related policies. All ASEAN countries which submitted VNRs included a significant number of environment-related policies. Of course, the VNRs do not provide evidence of whether the policies were sufficiently ambitious or effectively implemented. Nevertheless, the countries clearly made efforts to include environmental policies in their VNRs.

The second hypothesis that countries may have put a low priority on environmental policies was also not supported by the data. Environment-related policies accounted for a significant percent of the total number of policies in their VNRs (ranging from 21 to 50 percent). This does not necessarily mean that the countries’ governments in fact prioritized environment-related policies, since there are no data on actual implementation. Nevertheless, the countries clearly made efforts to give environmental policies a certain level of priority in their VNRs.

Moreover, this study found that the ASEAN countries did not include some of their other major environment-related policies in their VNRs, especially policies on air pollution and waste. Countries clearly have policies in these areas, but these areas were not highlighted in the SDGs, so countries may have overlooked them. This may be due to the fact that for most countries, most or even all policies may be somehow related to SDGs, and most countries have a very large number of policies in general. Therefore, it is inherently difficult to list all of them. SDG VNRs are a major reporting burden requiring inputs from many government departments.

Countries seemed to understand that the environment is related to a broad range of SDGs (not just the ones typically considered “environmental”—SDGs 12–15). Countries generally included environment-related policies among a broad range of SDGs, and some policies were listed under more than one SDG, although these efforts were not comprehensive or systematic. Countries listed the most environment-related policies under SDG 13, followed by SDGs 14 and 15.

The third hypothesis that environment-related policies were mainly small projects or programs, awareness-raising campaigns, capacity building, etc., was not supported. Many environmental policies mentioned in the VNRs appeared substantial, including national action plans and strategies as well as laws, regulations, specific infrastructure projects, tax/spending, monitoring/enforcement, etc., although again, the extent of actual implementation could not be assessed.

Regarding the fourth and fifth hypotheses, the results showed that the total number of environment-related polices and their share in the total number of SDG-related policies were not clearly correlated with GDP, per capita GDP, or VNR year. Some countries with lower GDP or lower GDP per capita had more environmental policies and/or a higher share of environment-related policies compared to countries with higher GDP or GDP per capita.

It is possible to speculate and develop hypotheses regarding the reasons for these results, but further research would be necessary to test them. One hypothesis is that countries made efforts to list policies relating to SDGs that were highlighted at the HLPF or related to the HLPF’s focus themes during the year their VNR was presented. This was also observed by Elder (2020) and Elder and Bartalini (2019). The countries also may have tried to highlight policies which are more substantial, newer, more interesting, and/or policies related to the government’s current political priorities. A country’s VNR could have included policies based on all of these possible reasons. The high percentage of environment-related policies suggests that there was some effort to highlight environmental efforts in the VNRs, regardless of their actual level of prioritization by the governments. It is also inherently difficult to align some kinds of policies with specific SDGs, especially broad national plans and strategies which cover a wide range of policy areas, for example, those relating to climate. Finally, developing countries might be using the VNRs to communicate with international development donors regarding their progress on specific issues of donor interest as well as build a case for additional financial assistance; this may partly explain the relatively high number of climate-related policies mentioned by the ASEAN countries.

## Conclusions

Overall, this study found that the ASEAN countries indicated a significant number of environment-related policies across a range of SDGs in their VNRs. Many of these policies appeared to be substantial, including national action plans and strategies, laws and regulations, and taxes and spending, not just small projects. Moreover, it was also observed that the countries are likely to be under reporting their environment-related policies. Therefore, it cannot be concluded that a lack of policies or insufficient prioritization of environmental SDG targets by ASEAN countries are the reason for their insufficient progress on the environmental dimensions of the SDGs, simply based on the information in the countries’ VNRs. Further research is needed on the level of ambition and extent of implementation of these environmental policies in order to understand the reasons for insufficient progress.

The dataset of ASEAN countries’ environment-related policies from their VNRs (ESM 2.1–2.9) is an important empirical value-added contribution of this study, and it is an essential basis for further research on the ASEAN countries’ environment-related SDG policies. To conduct any kind of policy analysis, including policies’ effectiveness, environmental impacts, and interlinkages, it is first necessary to identify the policies to analyze, which is not an easy task.

This study has several limitations. Most importantly, it does not address the effectiveness of these policies, or to what extent they were implemented. Second, the number of policies is not a good measure of effort, since the policies vary tremendously in terms of their magnitude and scope, from small programs like telephone hotlines to comprehensive national development strategies. Third, many or most “economic” and “social policies,” although not classified as “environmental,” are likely to have environmental effects, both positive and negative. Fourth, this study did not examine budgets because the VNRs included little related information. Fifth, this study only looks at government policies, not other stakeholders’ efforts. Sixth, sometimes it was difficult to determine whether a policy was related to the environment; in unclear cases, some additional checking was attempted, but additional information was not always available on the web or in English. Seventh, policies’ time frames were not systematically examined. Clearly some policies already existed before SDGs, but it is reasonable to count these, since they presumably contributed to the existing level of a country’s SDG achievement.

This study can contribute to further research in four major ways. First, this study’s environmental policy dataset could be used to as a basis to analyze more specific environment-related policy areas in ASEAN countries such as climate, energy, or water. This study would facilitate the identification of policies on a specific topic under more than one SDG, such as policies related to climate and circular economy. It would also help to identify policies which directly or indirectly influence environmental targets, particularly economic policies, which are not usually listed under SDGs like 13, 14, and 15.

Second, identification of the relevant policies, which is accomplished by this study’s dataset, is an essential step in analyzing policy effectiveness in all policy areas. Most discussions on SDGs still focus on achievement levels. From now, the focus of analysis should shift to the effectiveness of the implementation policies and the factors influencing their effectiveness. A policy dataset is necessary to start this analysis.

Third, the policy dataset can guide analysis of budget allocations, since presumably, most policies have associated budgets. Most countries have indicated plans to link or map their national plans and budgets with the SDGs, which would greatly facilitate more detailed analysis of countries’ SDG-related budgets. However, in cases where a budget map has not been prepared, it is necessary to start with a list of policies in order to search for the associated budgets.

Fourth, similar datasets of environment-related policies using a similar methodology could be developed for countries in other regions. This would facilitate cross-national and cross regional comparison of environment-related SDG policies, for example on climate, water, resource efficiency, circular economy, etc.

## Supplementary Information

Below is the link to the electronic supplementary material.

Supplementary file1 (DOCX 129 kb)

## Data Availability

The data used in this study are all publicly available. Electronic supplementary material is included with the main article.
