# Supplementary material for: ASEAN countries’ environmental policies for the Sustainable Development Goals (SDGs)
Source: Environ Dev Sustain. 2022 Jul 23:1–19. Online ahead of print. doi: 10.1007/s10668-022-02514-0 (PMC9307434; doi:10.1007/s10668-022-02514-0)
Supplement: Supplementary file 1 — Supplementary file1 (DOCX 129 kb) [file 10668_2022_2514_MOESM1_ESM.docx]

**Electronic Supplementary Material (1): Discussion of Individual Countries**

*Brunei Darussalam*

Brunei Darussalam’s VNR, published in 2020, addressed all SDGs. It is the most recent among ASEAN countries. It lists the highest total number of SDG policies, but the total number of environmental policies (65) is average, and the percentage directly related to the environment (24.1 percent) is the lowest (excluding Indonesia’s which does not cover every SDG).

Environment-related policies are listed mostly under the well-recognized environmental goals (12-15) and 11, while only a few are listed under 6 and 7. Unlike other ASEAN countries, no environmental policies are mentioned under SDG 2. Most countries did not list any environment-related policies under 5, but Brunei mentioned one on equal access to agricultural land. The only environment-related policy listed under SDG 9, the ‘Connected Smart Nation’ transport plan, focused on sustainability and energy efficiency. Overall, environment was not the VNR’s major focus, but Brunei may have more environmental policies which were not listed.

Like other ASEAN countries, policies under SDGs 13 and 14 may also apply to SDGs 7 and 6, respectively. Disaster Risk Reduction Plans could also be linked to SDG 1. Components of Brunei’s National Climate Policy could be listed under various SDGs due to its broad-ranging scope. Unlike other ASEAN countries, most policies in the VNR were repeated under all related SDGs, which shows more systematic thinking. Overall, Brunei Darussalam’s VNR was comprehensive but did not emphasize environmental issues.

*Cambodia*

Cambodia’s VNR, published in 2019, included all SDGs, but especially focused on the six highlighted at that year’s HLPF. The number and percent of environment-related policies in Cambodia’s VNR were about average for ASEAN countries. Many policies were not described in detail, so they were further checked to see if they addressed environmental issues. For example, Cambodia’s policy for women’s equality, Neary Rattanak IV, explicitly addresses women’s equal ownership of land and natural resources, so it should have been listed under SDG 5. Similarly, Cambodia did not list a policy addressing target 2.5 on genetic diversity of food, but the Agriculture Sector Strategic Development Plan under SDG 2 does address it.

Cambodia integrated environmental contents throughout the VNR and in relation to all SDGs more extensively compared to other ASEAN countries, and it has the highest percent (31) of total environmental policies which can apply to more SDGs than they were originally listed under. For example, Cambodia originally listed only five environmental policies under SDG 6, but many policies relating to water pollution and water resource management are listed under SDGs 11, 13 and 14, and were not originally also listed under SDG 6. Cambodia was the only ASEAN country which listed a policy related to the environment under every SDG except for 10, 16, and 17. It was also the only country to list an environmental policy under SDG 3. Moreover, many of Cambodia’s policies are large scale, long-term and/or national policies which can apply to many SDGs. Often these were only listed under one SDG so the VNR underrepresents the scope of Cambodia’s environment-related SDG policies.

*Indonesia*

Indonesia’s two VNRs, published in 2017 and 2019, only focused on the SDGs highlighted at those years’ HLPF, so key environmental SDGs (6, 7, 11, 12, 15) were not systematically addressed (although one policy on SDG 12 was mentioned). This probably explains why Indonesia had the lowest percentage of environmental policies among ASEAN countries (21.3 percent). Therefore, Indonesia’s VNRs significantly understated its environment related policies, since the country clearly had policies in areas such as water, renewable energy, SCP, biodiversity, forest conservation, etc. Indonesia was one of only three ASEAN countries to list policies under target 4.7 on education for sustainable development and SDG 17.

Among ASEAN countries, Indonesia had the highest percentage (37) of environmental policies which can apply to additional SDGs compared to their VNR’s original listing. For example, eight policies under SDG 13 address disaster preparedness which is also relevant to target 1.5. Likewise, various renewable energy policies for target 7.2 are listed under other SDGs. This may be partly because each VNR only focused on the SDGs highlighted at that year’s HLPF. This also makes it very difficult compare Indonesia with other ASEAN countries, which mostly addressed all SDGs in their VNRs.

*Lao PDR*

Lao PDR’s VNR, published in 2018, reviewed all SDGs, which are embedded in the Eighth National Socio-Economic Development Plan (NSEDP). Although Lao PDR has the second lowest number of environment-related policies among the ASEAN countries, the percentage related to the environment is slightly above average (45.5%). These policies are not evenly distributed throughout the SDGs; 23 are under SDGs 12-15, and 12 are under the others, including five under SDG 11. Thus, Lao PDR’s VNR included relatively more environmental policies compared to other ASEAN countries, even if it did not have policies for some environment-related targets. Major, broad policies were often listed under multiple SDGs, but this study still found some policies which applied to more SDGs than originally listed (22). Most were related to SDG 6, although they were originally listed under SDG 11 or were part of large, national climate policies. Thus, although Lao PDR reported a smaller number of environment related policies compared to the other ASEAN countries, there was still a significant emphasis on environmental concerns.

*Malaysia*

Malaysia’s VNR, published in 2017, covered all SDGs. The Eleventh Malaysia Plan 2016-2020 (11MP) incorporated most of them. While the total number of environment-related policies (66) is about average among ASEAN countries, Malaysia’s percentage of environmental policies out of its total policies is the highest (50.8 percent). The policies are not well distributed throughout the SDGs; 50 percent of them are under SDGs 14 and 15. Although the number of policies listed under SDGs 7, 9, and 11 is similar to other ASEAN countries, only one is listed under SDG 6, considerably less than most other ASEAN countries.

Malaysia also listed policies under multiple SDGs, though not systematically. Many policies listed under SDG 14 also apply to 6. Moreover, although Malaysia did not list any environmental policies under SDGs 1, 3, or 4, this study found policies related to these SDGs listed elsewhere. Overall, the percentage of policies which could apply to more SDGs than the original listing in the VNRs is about the same as other ASEAN countries. When considering repeated policies, environment related policies are more evenly distributed among the SDGs than the original listing in the VNR. Since Malaysia listed many large-scale policies without elaborating their contents, this study examined several in more detail, and found 6 which applied to additional SDGs.

*Philippines*

The Philippines published 2 VNRs (2016, 2019). The first focused mainly on overall national priorities, not progress on individual SDGs. The second addressed individual SDGs, but only the 6 highlighted at that year’s HLPF. Therefore, it is unsurprising that the country listed the second lowest number of total policies among ASEAN countries, and it is very difficult to compare the Philippines with the other ASEAN countries, which addressed all of the SDGs (except Indonesia, which addressed 12 SDGs in its 2 VNRs). It also has a slightly below average percentage of these policies which are directly related to the environment (35.9). Like Indonesia, this is partly because key environmental SDGs such as 6, 11, 12, 14, and 15 were not addressed at that year’s HLPF. Nevertheless, the real number of environmental policies could be higher than average if we assume that a similar percentage of environment related policies would have been listed if the other SDGs had been addressed.

It is difficult to conclude much about the overall distribution of environmental policies among the SDGs because the VNR did not review all of SDGs. Nevertheless, like Indonesia, in total, a high percentage (30 percent) of environmental policies can be applied to more SDGs than they are listed under, the third highest level among ASEAN countries. Environmental policies were listed under only two of the six SDGs in the VNR (8 and 13). However, 6 policies under SDG 13 can also be applied to target 1.5, and 3 can also be applied to target 7.3. The Philippines listed more policies under SDGs 8 and 13 than other ASEAN countries. Also, the repetition of some policies under SDGs 8 and 13 indicates a recognition of synergies between climate and the economy.

*Singapore*

Singapore’s VNR, published in 2018, addressed all SDGs. As expected from ASEAN’s most developed country, its VNR was comprehensive, linking policies with achievements. Singapore listed the second highest number of total policies in its VNR, after Brunei Darussalam, and the highest number of policies directly related to the environment. However, the environment-related policies’ share of total policies (44.7 percent) was only the third highest in ASEAN. Overall, Singapore’s VNR was substantial and comprehensive, including many small policies as well as broad national strategies.

The distribution of environmental policies among SDGs in Singapore’s VNR was similar to the other ASEAN countries, and 33 percent of them are listed in SDGs other than 12-15. Like most ASEAN countries, no environmental policies are listed under SDGs 1, 3, 4 and 5, but unlike most other countries Singapore surprisingly does not list any environmental policies under SDG 8. Moreover, SDG 8 is one of the few areas with little policy content in Singapore’s VNR. However, like Indonesia, Singapore listed some environment related policies under SDG 17.

The number of policies which could apply to more SDGs than listed in the VNR is similar to the other ASEAN countries. Especially, some policies could be additionally listed under SDGs 3, 6, 7 and 9. For example, Singapore listed many policies on hazardous waste and pollution under SDG 12 which could also could have been listed under target 3.9 (deaths and illnesses from pollution). Also, while Singapore listed many policies under SDG 6, other policies could also have been listed there, especially the water efficiency policies under SDG 12. Singapore listed only one environmental policy under SDG 9, but eight policies under SDG 11 also address sustainable industrialization and could have been listed under SDG 9. Overall, Singapore listed a substantial number of environment related policies in its VNR.

*Thailand*

Thailand’s VNR, published in 2017, addressed all SDGs, which were incorporated into the 20-Year National Strategy Framework and the 12th National Economic and Social Development Plan (2017-2021). These were based on the core principle of Sufficiency Economy Philosophy (SEP), which is integrated throughout the VNR.

Thailand’s VNR is broad-ranging with many policies. The total number of environment-related policies as well as the percentage of policies related to the environment are about average among ASEAN countries. The environment-related policies are well distributed throughout the SDGs, including under SDGs 1, 4, and 17, in contrast to many other ASEAN countries. About 40 percent of the environmental policies are listed under SDGs other than 12-15. Thailand has the most environmental policies of all ASEAN countries under SDGs 1, 2, 4 and 9. Especially interesting is the emphasis in SDG 2 on sustainable and environmentally friendly agriculture, a topic which is surprisingly absent in many of the ASEAN countries’ VNRs.

In contrast to the other ASEAN countries, few of Thailand’s environmental policies were found to apply to additional SDGs compared to the ones they were listed under originally (about 14 percent). The reason is not clear. Thailand may have more accurately matched its policies with the SDGs compared to other countries, or it may have included fewer wide-ranging policies covering multiple policy areas. Overall, the number and distribution of environment-related policies in Thailand’s VNR is similar to the other countries.

*Viet Nam*

Viet Nam’s VNR, published in 2018, addressed all SDGs. Vietnam has developed its own national SDG plan, the ‘National Action Plan for Implementation of the 2030 Agenda.’ Vietnam has both a high number of total policies listed in its VNR as well as a high percentage of environmental policies (50.2), the second highest in ASEAN.

The distribution of environmental policies among SDGs is similar to other countries in ASEAN, and about 35 percent of environmental policies are in SDGs other than 12-15. No environmental policies were listed under SDGs 1, 3, 4, or 5, but many policies were listed under SDGs 6 and 8, suggesting that Vietnam has recognized the importance of synergies between the environment and economy. Some policies were applicable to more SDGs than they were listed under. Four also address target 1.5; the Law on Natural Disaster Prevention and Control listed under SDG 13 was directly related, while the other three (National Strategy on Climate Change, National Strategy for Environmental Protection, and National Strategy for Sustainable Development) were broad national strategies covering many policy areas, not just disasters and resilience. These broad strategies were not listed under all the SDGs that they are related to. Overall, Viet Nam’s VNR reported a significant number of environment-related policies distributed among many, though not all, SDGs.

**Electronic Supplementary Material (2): Dataset of ASEAN Countries’ Environment Related Policies in their SDG VNRs**

This supplementary material is the dataset of tables which list the environment-related policies included in each country’s VNR. The starting point of this dataset is the comprehensive list of the ASEAN countries’ SDG policies which was originally compiled by (Elder 2020) based on the countries’ VNRs. In some cases, the text was edited in this study to make it more concise. The dataset in this appendix identified the environment-related policies from the policies listed in Elder 2020, based on the criteria in (Elder and Olsen 2019). In addition, the current dataset identified which policies were repeated. The middle column in the tables indicates the policies which were directly mentioned in the VNRs, and bold text indicates which policies were repeated by the countries in their VNRs. The right-hand column indicates which other SDGs the policy applies to based on the authors’ assessment. Policies listed in italics were researched in further detail, as their relation to the environment was not clear from the VNR.

List of Tables

**Table ESM 2.1: *Environment-related policies in Brunei Darussalam’s VNR***

**Table ESM 2.2: *Environment-related policies in Cambodia’s VNR***

**Table ESM 2.3*: Environment-related policies in Indonesia's 2017 and 2019 VNRs***

**Table ESM 2.4*: Environment-related policies in Lao PDR’s VNR***

**Table ESM 2.5*: Environment-related policies in Malaysia’s VNR***

**Table ESM 2.6*: Environment-related policies in the Philippines’ VNR***

**Table ESM 2.7*: Environment-related policies in Singapore’s VNR***

**Table ESM 2.8*: Environment-related policies mentioned in Thailand’s VNR***

**Table ESM 2.9*: Environment-related policies in Viet Nam’s VNR***

Table ESM 2.1: Environment-related policies in Brunei Darussalam’s VNR

| SDG | Policies which were directly mentioned | Other Applicable SDG targets |
| --- | --- | --- |
| 1 | Temporary housing shelters and assistance for disaster relief and home improvements |  |
| 2 | [None listed] |  |
| 3 | [None listed] |  |
| 4 | [None listed] |  |
| 5 | Equal ownership of land (Brunei Land Code (Cap. 40)) and equal access to agricultural land, such as the Agricultural Development Area and the Rural Agricultural Area |  |
| 6 | 1. Implementing a water tariff system and Unified Smart Metering System, with new rates using a tiered structure for residential, commercial, and raw water 2. Wastewater treatment service is provided at no cost to the public 3. Ministerial, NDP projects provide sanitation access to 93% of the population |  |
| 7 | 1. Ministry of Energy (ME) conducts promotion of plans and programmes related to Energy Efficiency and Conservation, Renewable Energy **(in SDGs 7 & 13)** 2. 30 MW solar farm at Sungai Akar is planned 3. Bilateral power interconnection project with Malaysia is planned to support RE |  |
| 8 | 1. Ministry of Primary Resources and Tourism (MPRT)’s Strategic Plan 2016-2020 2. The Tourism Development Department promotional packages for niche, sustainable activities 3. The Safety, Health and Environment National Authority (SHENA) (2017), regulates and enforces matters of workplace safety and health, environment, and radiation. |  |
| 9 | *Ministry of Transport and Infocommunications Strategic Plan 2020-2025, “Connected Smart Nation”* |  |
| 10 | [None listed] |  |
| 11 | 1. National Land Use Master Plan, District Plans, Local Plans and Planning Schemes 2. *Piawaian Brunei Darussalam 12:2017 - Building Guidelines and Requirements* 3. Environmental Protection & Management Order (EPMO) 2016. Activities with environmental impacts are required to submit Written Notification & EIAs/Environmental Management & Monitoring Plans (EMMP) **(in SDGs 11 & 15)** 4. Hazardous Waste (Control of Export, Import and Transit) Order (HWO) 2013 5. The Department of Environment, Parks and Recreation (JASTRe) policies 6. Solid Waste Management Strategy 7. Air pollution related action plans 8. River quality monitoring by the Sewerage Laboratory of sewage plant discharge 9. Mahkota Jubli Emas Park (2017) for recreational activities 10. Closure of selected roads in the capital every Sunday for the Bandarku Ceria 11. Integrated flood risk reduction approach 12. Strategic National Action Plan for Disaster Risk Reduction 13. Disaster Preparedness Coordinator programme trains community leaders | D: 3.9  H: 6.3  K: 1.5  L: 1.5  M: 1.5 |
| 12 | 1. Various policies and initiatives to reduce plastic use 2. Excise duties on plastic products were increased in 2017 3. The Municipal Department enforces proper waste disposal especially in public areas 4. Various policies on sustainable use of natural resources 5. Regulations to prevent overfishing include zoning systems, MSY, allocation of fishing licenses, specific allowable types of fishing gear, and ban on trawlers **(in SDGs 12 & 14)** 6. Measures to promote environmental awareness were mentioned **(in SDGs 12 & 13)** |  |
| 13 | 1. Brunei National Climate Policy (BNCP) 2. Policies to promote renewable energy development **(in SDGs 7 & 13)** 3. Smart electricity tariff system and smart meters to promote energy efficiency 4. Energy management in government and commercial sectors 5. New electricity tariffs for commercial and industry sectors 6. Energy Efficiency and Conservation (EEC) Building Guidelines for Non-Residentials 7. Ministry of Energy actions include oil/gas GHG emissions abatement projects 8. Forest plantation programme 9. Measures to promote environmental awareness were mentioned **(in SDGs 12 & 13)** 10. Online carbon footprint calculator for the public | A: 4.7; 7.2; 9.1, .2, .4  C: 7.3  D: 7.3  E: 7.3  F: 7.3 |
| 14 | 1. Fisheries Order 2009 governs marine biodiversity protection **(in SDGs 14 & 15)** 2. Marine Protected Areas (MPAs) 2015 3. Enforcing zoning systems with license quotas for fishing **(in SDGs 12 & 14)** 4. Enforcement of allowable types of fishing gear **(in SDGs 12 & 14)** 5. Banning of trawlers is expected to take effect by 2021 **(in SDGs 12 & 14)** 6. Banning any catching of endangered species from Brunei Darussalam’s waters and their sale in domestic markets, as well as import and trade of shark products 7. National Plan of Action to Prevent, Deter and Eliminate Illegal, Unreported and Unregulated Fishing 2011 8. Fishery Limits Act (Cap. 130) **(in SDGs 14 & 15)** 9. Maximum Sustainable Yield (MSY) **(in SDGs 12 & 14)** | A: 6.6  B: 6.6 |
| 15 | 1. Forest Act, enacted in 1934, revised 2013 (Cap. 46) 2. National Forestry Policy 1989 3. Wild Life Protection Act 1978 4. Wild Fauna and Flora Order 2007 5. Land Code 6. Land Acquisition Act 7. Antiquities and Treasure Trove Act 8. Fishery Limit Act (Cap. 130) **(in SDGs 14 & 15)** 9. Fisheries Order 2009 **(in SDGs 14 & 15)** 10. Town and Country Planning Act 11. Environmental Protection and Management Order 2016 **(in SDGs 11 & 15)** 12. Pulau Selirong Forest Reserve, Ulu Temburong National Park and Berakas Forest Reserve, were dedicated to the Queen’s Commonwealth Canopy 13. The government regulates the entry of potential invasive species 14. Brunei Selection Felling System (BSFS), 1986, aims to enhance timber productivity 15. Promotion of research to determine and identify new and unknown species |  |
| 16 | [None listed] |  |
| 17 | [None listed] |  |

Source: (Government of Brunei Darussalam, 2020) as compiled by (Elder, 2020).

Table ESM 2.2: Environment-related policies in Cambodia’s VNR

| SDG | Policies which were directly mentioned | Other Applicable SDG targets |
| --- | --- | --- |
| 1 | *National Social Protection Policy Framework 2016-2025* |  |
| 2 | Agriculture Sector Strategic Development Plan 2014-2018 | 6.5 |
| 3 | *Health Strategic Plan 2016-2020* |  |
| 4 | *National Education 2030 Roadmap for CSDG4* |  |
| 5 | 1. *The Ministry of Women’s Affairs’ five-year Strategic Plan for Gender Equality and Women’s Empowerment 2014-2018, Neary Rattanak IV* 2. The Strategic Plan on Gender, Climate Change, Green Growth, and Disaster Risk Management have been integrated into the Climate Change Strategic Plan |  |
| 6 | 1. Integrated Water Resource Management implementation process 2. National Strategic Plan on Water and Rural Sanitation 2011-2025 3. First National Action Plan for Rural Water Supply, Sanitation and Hygiene 4. Second National Action Plan for Rural Water Supply, Sanitation and Hygiene 5. Ministry’s Rural Development Strategic Plan 2019-2023 |  |
| 7 | The Ministry of Mines and Energy is updating the Power Development Master Plan and developing renewable energy policy |  |
| 8 | “China Ready” policy (2016) encouraging businesses to offer ecotourism options |  |
| 9 | *Industrial Development Policy (IDP) 2015-2025, about 100 measures* | 6.1 |
| 10 | [None listed] |  |
| 11 | 1. The Ministry of Environment (MOE) has transferred 6 functions to sub-national level on the collection of solid waste, the management of drainage system, and natural resource protection 2. MOE took actions including monitoring & inspecting pollution sources, piloting reporting system, and inspecting environmental pollution violence 3. The MOE worked to raise awareness on plastic bag use 4. The Ministry of Public Works and Transport prepared a master plan on waste water & sewage system management for major cities as a response to the RS-IV. 5. *National Policy on Spatial Planning* 6. Sub-Decree on Municipal Solid Waste Management **(in SDGs 11 & 12)** 7. Sub-Decree on Social Environmental Fund **(in SDGs 11 & 12)** 8. Sub-Decree on the Management of Electrical and Electronic Waste **(in SDGs 11 & 12)** 9. Sub-Decree on Plastic Bag Management **(in SDGs 11 & 12)** 10. Sub-Decree on Management of Sewage System & Water Treatment **(in SDGs 11 & 12)** | A: 6.3  B: 6.3  D: 6.3  J: 6.3 |
| 12 | 1. The MOE monitors air quality at 7 sites to check the acidic levels 2. Sub-Decree on Municipal Solid Waste Management **(in SDGs 11 & 12)** 3. Sub-Decree on Social Environmental Fund **(in SDGs 11 & 12)** 4. Sub-Decree on the Management of Electrical and Electronic Waste **(in SDGs 11 & 12)** 5. Sub-Decree on Plastic Bag Management **(in SDGs 11 & 12)** 6. Sub-Decree on Management of Sewage System & Water Treatment **(in SDGs 11 & 12)** 7. SCP is integrated into the Environment and Natural Resources Code under the Environmental Management and Sustainability Mechanism |  |
| 13 | 1. The NCSD established Climate Change Technical Working Group for SDG 13 2. Climate change is integrated in the Rectangular Strategy IV 2018-2023 **(in SDGs 13 & 14)** 3. Climate change is integrated into the National Strategic Development Plan 2019-2023 4. UNFCC Nationally Determined Contribution, including 13 priority actions 5. National Environment Strategy and Action Plan **(in SDGs 13 & 14)** 6. Cambodia Climate Change Strategic Plan 2014-2023 **(in SDGs 13 & 14)** 7. Environment and Natural Resources Code has been finalized 8. Technical guidelines are gradually being introduced in concerned ministries e.g. to climate-proof water and road infrastructure, or improve the capacities of health professionals to prevent and treat climate-sensitive diseases | B: 3.9; 6.3, .6  D: 6.5, .6; 7.2, .3  E: 6.5  F: 1.5; 2.5; 3.9; 4.7; 6.2, .3, .5, .6; 7.2, .3; 8.9  H: 3.9 |
| 14 | 1. National Strategic Plan on Green Growth 2013-2030 2. Climate change is integrated in the Rectangular Strategy IV 2018- 2023 **(in SDGs 13 & 14)** 3. Cambodia Climate Change Strategic Plan 2014-2023 **(in SDGs 13 & 14)** 4. National REDD+ Strategy **(in SDGs 14 & 15)** 5. Sub-Decree on Water Pollution Control 1999 6. National Environment Strategy and Action Plan 2016-2023 **(in SDGs 13 & 14)** 7. National Adaptation Plan Financing Framework & Implementation Plan in 2017 8. Strategic Planning Framework for Fisheries 2010-2019 | A: 8.4  E: 6.3  H: 6.6 |
| 15 | 1. Established and implemented 6 policies, strategies and regulations on ecosystem services in 2015. In 2016, 2017 and 2018, the number of policies, strategies and regulations issued and implemented exceeded the CSDG targets 2. National Protected Area Strategic Management Plan 2017-2031 3. National REDD+ Strategy 2018-2028 **(in SDGs 14 & 15)** 4. Cambodia continues to strengthen and enhance environmental education | D: 4.7 |
| 16 | [None listed] |  |
| 17 | [None listed] |  |
| 18 | [None listed] |  |

Source: (Kingdom of Cambodia, 2019) as compiled by (Elder, 2020).

Table ESM 2.3: Environment-related policies in Indonesia's 2017 and 2019 VNRs

| SDG* | Policies which were directly mentioned | Other Applicable SDG targets |
| --- | --- | --- |
| 1 (2017) | 1. The number of natural disaster victims who received government assistance, increased from 66,625 in 2010 to 200,000 in 2014. Staffing and related training increased from 100 persons in 2010 to 295 persons in 2014. 2. Indonesia Electrification Program (Program Indonesia Terang/PIT) 2016 | B: 7.2 |
| 2  (2017) | 1. Draft food and nutrition action plan and a Food Security and Vulnerability Atlas 2. National Community Empowerment Program for Healthy and Smart Generation (PNPM-GSC) | A: 6.1  B: 6.2 |
| 3 | [None listed] |  |
| 4 | WASH in School (improving access to clean water, sanitation, and hygiene) | 6.1, .2 |
| 5 | [None listed] |  |
| 6 | [None listed] |  |
| 7 | [None listed] |  |
| 8 | 1. Drastically cut fuel subsidies to fund infrastructure development 2. Develop 10 Tourism Priority Destinations, increasing sustainability of Bali tourism; strengthening 11 potential destinations; developing ecotourism and marine tourism |  |
| 9  (2017) | 1. Lamong Bay Terminal (innovative infrastructure project that adopts environmentally friendly principles) 2. Sustainable Financial Roadmap (new sustainability standard for financial services) |  |
| 10 | [None listed] |  |
| 11 | [None listed] |  |
| 12 | The company performance rating program (Proper) assesses the level of companys’ environmental management |  |
| 13 | **DRR (Disaster Risk Reduction)**   1. Disaster Management Plan (RPB) in all provinces and 30% at the district/city level 2. DRR policies are internalized into 2015-2019 Government Work Plan 3. Disaster risk research and mappings were conducted in provinces & districts/cities 4. Established Tangguh Bencana Village in 594 villages in 186 districts/cities. A Tangguh Bencana Village has a capability to deal with disasters and recover immediately 5. The Government supports capacity building on regional disaster management for regional government officials in collaboration with the Training Agency 6. The Government provides support for local governments and communities in improving disaster awareness culture and preparedness 7. Disaster Management Priority Program focuses on 136 priority districts   **Climate Change Adaptation**   1. 15 pilot areas for the National Action Plan for Climate Change Adaptation (RAN-API) 2. The API-PRB convergence process aims to reduce adverse impacts of climate disasters 3. Conservation Agriculture programs in some provinces to assist climate adaptation 4. Farmers Field Schools program   **Climate Mitigation**   1. PR No. 61 (2011) on Greenhouse Gas Action Plan **(in SDGs 13 & 17)** 2. PR No. 71 (2011) on GHG emissions, with emphasis on climate mitigation **(in SDGs 13 & 17)** 3. Government Regulation (GR) 46 on Strategic Environmental Assessment (SEA) 4. Forest and peat land mitigation actions (6 examples). 5. Mitigation actions in the agriculture sector (3 examples). 6. Mitigation actions by the Ministry of Energy and Mineral Resources (5 activities). 7. Four mitigation activities for energy conservation/diversification in the industry sector. 8. Policies in the form of mitigation actions in the transportation sector. 7 examples 9. Policies in the form of mitigation actions in the waste management sector 10. Indonesia Climate Change Trust Fund 2009 11. ‘Low Carbon Development Initiatives’ implemented 2 pilot projects. (1) (ICCTF) project in Central Java which invests in bio-digesters to produce compost; (2) the West Papua provincial government signed a sustainable province commitment at International Conference on Biodiversity, Ecotourism and Creative Economy held in Manokwari, West Papua | A-G: 1.5  I: 1.5  J: 2.4  K: 2.4  P: 2.4  Q: 7.2, .3  R: 7.3; 9.4  S: 7.2; 9.1  T: 6.3  V: 7.2; 8.9; 9.4 |
| 14  (2017) | 1. As of 2016 Indonesia has declared 165 Marine Protected Areas (MPAs) 2. Ocean Act 32 of 2014, the basis for Indonesia’s marine spatial planning 3. National Marine Spatial Plan (RTRLN), supports integrated and sustainable use of marine and coastal areas 4. Marine and Fisheries Affairs Ministerial Regulation No. 18/2014 established 11 Fisheries Management Areas (Wilayah Pengelolaan Perikanan/WPP). A Fisheries Management Plan must be developed by each WPP **(in SDG 14 2017 & 2019)** 5. Act No. 31/2004 jo Act No. 45/2009 aims to reduce IUU fishing 6. The establishment of an information system for vessels 7. Management Effectiveness for Marine Protected Area (E-MPA) | A: 6.6  C: 6.6 |
| 14  (2019) | 1. During 2015-2018, marine protected areas increased from 17.3 to 19.3 million ha 2. Fisheries Management Plans (RPP) for all WPP have been determined through the Ministerial Decree of Marine & Fisheries Affairs **(in SDG 14 2017 & 2019)** |  |
| 15 | [None listed] |  |
| 16 | [None listed] |  |
| 17 | **Innovative Financing**   1. The KPBU is a type of infrastructure financing. 19 KPBU projects include toll road construction, and energy, telecommunication, and water provision. One successful case is Drinking Water Provision System in Umbulan 2. PT SMI issued the first green bond in Indonesia in 2018 3. Financial Services Authority’s (OJK) comprehensive Sustainable Finance Roadmap 4. In July 2018, published a book explaining fikih zakat on SDGs (legal basis for Muslim’s contribution to SDGs), the first achievement of Islamic financing. The guideline is implemented through a blended finance scheme to build a Micro Hydro Power Plant in Jambi Province in Sumatera   **Climate Action**   1. Climate action policy started from PR 61 (2011) on Greenhouse Gas Action Plan and PR 71 (2011) on GHG Emission. **(in SDGs 13 & 17)** 2. Bappenas’ National Action Plan on Climate Adaptation 2014 3. MR 7 (2018) instructing subnational governments to incorporate SEA in their RPJMD and spatial plan of RTRW | A: 6.1; 9.1 |

* Note: SDGs with the designation (2017) indicate policies from the 2017 VNR, while policies under the other SDGs are from the 2019 VNR. The one exception is SDG 14; most policies were listed in the 2017 VNR, while the 2019 VNR repeated 2 of the previously listed environmental policies and indicated significant implementation progress.

Source: (Republic of Indonesia, 2017) (Republic of Indonesia, 2019) as compiled by (Elder, 2020).

Table ESM 2.4: Environment-related policies in Lao PDR’s VNR

| SDG | Policies which were directly mentioned | Other Applicable SDG targets |
| --- | --- | --- |
| 1 | [None listed] |  |
| 2 | 1. Agricultural Development Strategy 2020 2. *National Nutrition Strategy and Plan of Action* |  |
| 3 | [None listed] |  |
| 4 | [None listed] |  |
| 5 | [None listed] |  |
| 6 | 1. Updated Water and Water Resources Law 2017 2. Upscaling pilot initiatives in Champasak and Sekong provinces to expand rural sanitation 3. National River Basin Development Strategy 2030 and Five-Year Action Plan |  |
| 7 | Renewable Energy Development Strategy |  |
| 8 | [None listed] |  |
| 9 | Lao PDR Road Sector II Project (part of national program to build climate-resilient roads) |  |
| 10 | [None listed] |  |
| 11 | 1. Vientiane Sustainable Urban Transport Project was approved in 2015 2. 8^th^ NSEDP includes programmes to enhance urban water & sanitation, improve public governance & admin., and establish “Green & Clean Towns” (incl. climate-smart planning) 3. The Department of Housing and Urban Planning, and the Ministry of Public Works and Transportation have adopted an urban sector strategy. It includes strategies on solid waste, water sector investment and urban sanitation 4. The Ministry of Natural Resources and Environment (MoNRE) is developing 46 “Clean and Beautiful Guidelines” to promote environmental quality 5. MoNRE is proposing the National Land Use Master Plan to the National Assembly | A: 9.1  B: 6.1, .2  C: 6.2 |
| 12 | 1. The efficient use and management of natural resources is a priority in 8^th^ NSEDP 2. Ecotourism is a national priority in 8^th^ NSEDP 3. Lao PDR Action Plan to implement the 2016 Pakse Declaration on ASEAN Roadmap for Strategic Development of Ecotourism Clusters & Tourism Corridors 4. Green Growth National Steering Committee established in 2016 to develop the National Green Growth Strategy 5. MoNRE is in the process of identifying Green Procurement guidelines | B: 8.9  D: 8.4 |
| 13 | 1. National law on Intended Nationally Determined Contribution passed in 2016 2. National Climate Change Strategy 2010 **(in SDGs 13 & 15)** 3. Forestry Strategy to the Year 2020 (2005) **(in SDGs 13 & 15)** 4. Renewable Energy Development Strategy 2011 **(in SDGs 13 & 15)** 5. Sustainable Transport Development Strategy 2010 **(in SDGs 13 & 15)** 6. Climate Change Action Plan of Lao PDR for 2013-2020 **(in SDGs 13 & 15)** 7. National Adaptation Programme of Action 2009 **(in SDGs 13 & 15)** 8. Investment and Financial Flows to Address Climate Change in Energy Agriculture and Water Sectors 2015 | B: 2.4; 6.1, .2, .3  D: 7.2  E: 9.1  H: 6.6 |
| 14 | [None listed] |  |
| 15 | 1. Prime Minister’s Order No. 15, 2016, bans logging from production forests, implementing and testing more participatory models of forest management 2. National Climate Change Strategy 2010 **(in SDGs 13 & 15)** 3. Forestry Strategy to the Year 2020 (2005) **(in SDGs 13 & 15)** 4. Renewable Energy Development Strategy 2011 **(in SDGs 13 & 15)** 5. Sustainable Transport Development Strategy 2010 **(in SDGs 13 & 15)** 6. Climate Change Action Plan of Lao PDR for 2013-2020 **(in SDGs 13 & 15)** 7. National Adaptation Programme of Action 2009 **(in SDGs 13 & 15)** 8. National REDD+ Strategy (to be approved by June 2018) 9. Forest Resource Development Fund 10. Certification model testing has started. Introduce important forest governance reforms including certification and timber legality assurance systems |  |
| 16 | [None listed] |  |
| 17 | [None listed] |  |
| 18 | [None listed] |  |

Source: (Lao PDR, 2019) as compiled by (Elder, 2020).

Table ESM 2.5: Environment-related policies in Malaysia’s VNR

| SDG | Policies which were directly mentioned | Other Applicable SDG targets |
| --- | --- | --- |
| 1 | [None listed] |  |
| 2 | 1. *National Agrofood Policy (NAP)* 2. Malaysia adopted a series of certification schemes of good agricultural practices, such as Malaysia Good Agricultural Practices (myGAP), Malaysia Organic (myOrganic), and Malaysia Sustainable Palm Oil (MSPO). Malaysia also has been proactive in maintaining genetic diversity and undertaking research in climate-resistant crops and farmed animals |  |
| 3 | [None listed] |  |
| 4 | [None listed] |  |
| 5 | [None listed] |  |
| 6 | *11MP Strategic Thrusts 1, 4 and 5* **(in SDGs 6, 7, 11 & 13)** |  |
| 7 | 1. *11MP Strategic Thrusts 4 and 5* **(in SDGs 6, 7, 11 & 13)** 2. National Renewable Energy Policy and Action Plan 2009 3. National Green Technology Policy 2009 4. National Energy Efficiency Action Plan 2015 |  |
| 8 | 11MP Game Changer “Embarking on Green Growth” **(in SDGs 8, 9, 12, 14 & 15)** |  |
| 9 | 1. 11MP Game Changer “Embarking on Green Growth” **(in SDGs 8, 9, 12, 14 & 15)** 2. Sustainable consumption and production initiatives 3. Government Green Procurement 4. SCP in Education 5. Recycling rate for solid waste 6. National Ecotourism Plan 2016-2025 **(in SDGs 9 & 12)** 7. Malaysian Carbon Reduction and Environmental Sustainability Tool (MyCREST) | D: 4.7  F: 8.9 |
| 10 | [None listed] |  |
| 11 | 1. *11MP Strategic Thrusts 1, 2, 4 and 5* **(in SDGs 6, 7, 11 & 13)** 2. *National Physical Plan 3* 3. *National Urbanisation Policy 2* 4. *Competitive Cities Master Plans* 5. National Environmental Health Action Plan **(in SDGs 11 & 12)** 6. National Solid Waste Management Policy **(in SDGs 11 & 12)** | C: 6.3, .5; 7.2, .3; 8.9; 9.1, .2, .4  E: 3.9 |
| 12 | 1. 11MP Game Changer “Embarking on Green Growth” **(in SDGs 8, 9, 12, 14, 15)** 2. National Ecotourism Plan 2016–2025 **(in SDGs 9 & 12)** 3. National Policy for Biological Diversity 2016–2025 4. National Environmental Health Action Plan **(in SDGs 11 & 12)** 5. National Solid Waste Management Policy **(in SDGs 11 & 12)** 6. Solid Waste and Public Cleansing Management Corporation Strategic Plan 2014–2020 7. Department of National Solid Waste Management Strategic Plan 2016–2020 8. *Construction Industry Transformation Programme 2016–2020* | H: 9.2 |
| 13 | 1. *11MP Strategic Thrusts 2, 4 and 5* **(in SDGs 6, 7, 11 & 13)** 2. National Policy on Climate Change 2009 3. *National Urbanisation Policy 2006–2020* 4. *National Physical Plan 3* 5. National Green Technology Policy 2009 | B: 1.5; 2.4; 4.7; 7.2, .3; 9.1, .2  D: 9.1  E: 9.4 |
| 14 | 1. 11MP Game Changer “Embarking on Green Growth” **(in SDGs 8, 9, 12, 14,15)** 2. National Plan of Action for the Coral Triangle Initiative 2009 3. National Policy for Biological Diversity 2016-2025 4. National Oil Spill Contingency Plan 5. National Coastal Zone Physical Plan 6. National Ocean Policy 2011–2020 7. National Plan of Action to Prevent, Deter and Eliminate IUU Fishing 8. National Plan of Action for Management of Fishing Capacity in Malaysia 2014–2018 9. Malaysia has established 63 marine protected areas covering 16,492.92 km^2^ 10. A successful Mangrove Planting Programme was initiated in 2005 11. An extensive network of marine monitoring stations has been established 12. Programmes are being implemented to ensure sustainable fisheries and aquaculture 13. Ecosystem Approach to Fisheries Management to improve livelihoods and food security in coastal communities by sustainable fisheries and poverty reduction initiatives by 2020 14. State governments have implemented ‘No Plastic Bag’ campaigns to reduce pollution 15. Good Agriculture Practice (MyGAP) certification 16. Code of Practices for Seaweed Cultivation 17. Good Aquaculture Practice (GAqP) – Aquaculture Farm General Guidelines | B: 6.6  D: 6.6  E: 6.6  I: 6.6  J: 6.6  K: 6.6 |
| 15 | 1. 11MP Game Changer “Embarking on Green Growth” **(in SDGs 8, 9, 12, 14 & 15)** 2. National Policy for Biological Diversity 2016–2025 3. National Tiger Conservation Action Plans 2008–2020 4. Elephant Conservation Action Plans 2013–2022 5. National Forestry Policy 1992 6. National Action Plan on Peatlands 2011 7. Second National Mineral Policy 2009 8. Wildlife Conservation Act 2010 9. National Parks Act 1980 10. Biosafety Act 2007 11. Malaysia is drawing up a National Red Data List for threatened species and implementing innovative methods to combat poaching and illegal wildlife trade 12. National Conservation Trust Fund for Natural Resources 13. 1Malaysia Biodiversity Enforcement Operation Network operating in Taman Negara 14. Tapir Conservation Action Plan is being formulated 15. Central Forest Spine covers an area of 18,866 hectares of Permanent Forest Reserves in Peninsular Malaysia; Heart of Borneo covers 20 million hectares 16. Sustainable forest management and the Malaysian Timber Certification Scheme |  |
| 16 | [None listed] |  |
| 17 | [None listed] |  |

Source: (Government of Malaysia, 2017) as compiled by (Elder, 2020).

Table ESM 2.6: Environment-related policies in the Philippines’ VNR

| SDG | Policies which were directly mentioned | Other Applicable SDG targets |
| --- | --- | --- |
| 4 | [None listed] |  |
| 8 | 1. “National Spatial Strategy” identifies geographic development challenges & opportunities. Vulnerability reduction puts in place disaster risk resilience measures 2. Green Jobs Act 2016 **(in SDGs 8 & 13)** 3. National Green Jobs Human Resource Development Plan 4. Philippine Sustainable Consumption and Production Action Plan **(in SDGs 8 & 13)** 5. Tourism Act of 2009 promotes sustainable tourism. 6. National Tourism Development Plan 2016-2022 and National Ecotourism Strategy and Action Plan 2013-2022 7. The Securities and Exchange Commission will require Sustainability Reports for Publicly Listed Companies **(in SDGs 8 & 13)** | A: 1.5 |
| 10 | [None listed] |  |
| 13 | 1. Climate Change Act of 2009 established the Climate Change Commission (CCC) 2. National Disaster Risk Reduction and Management Act of 2010 3. People’s Survival Fund Act of 2013, financial support for local adaptation projects 4. Sectoral and framework plans updated to incorporate climate change and disaster risk parameters such as the Agriculture and Fisheries Modernization Plan, Philippine Energy Plan, Environment and Natural Resources Framework Plan, and river basin master plans 5. At the local level, Comprehensive Land Use Plans and Comprehensive Development Plans are also made climate and disaster risk-informed 6. The Cabinet Cluster on Climate Change Adaptation and Mitigation, and DRR (2011 and strengthened in 2017) to lead the effective coordination, harmonization, and complementation of policies and programs 7. CCC Resolution 2019-001 to harmonize and integrate efforts of sectors and stakeholders (issued by the National Climate Risk Management Framework) 8. Energy Efficiency and Conservation Act of 2019 9. Green Building Code of 2016 10. GHG Inventory Management and Reporting System 2014 11. National Integrated Climate Change Database and Information Exchange System 12. Green Jobs Act of 2016 **(in SDGs 8 & 13)** 13. Securities and Exchange Commission Memorandum Circular No.4 s. 2019 that provides the Sustainability Reporting guidelines **(in SDGs 8 & 13)** 14. Working on the completion of high-resolution multi-hazard and risk maps 15. National Color-Coded Agriculture Guide Map 2017 which contains projected rainfall and temperature information 16. Project NOAH partnership between the academe and government to provide weather information 17. Climate Budget Tagging System to track and monitor climate change-related expenditures 2015 18. Risk Resiliency Program (focuses on priority climate-vulnerable provinces) 19. Community-based Early Warning System 20. Green, Green, Green program to promote the development of public open spaces and create greener, and more sustainable cities 21. Public Utility Vehicle Modernization Program 22. National Ecolabelling Program 23. Government Energy Management Program 24. Green Public Procurement 25. Waste management programs such as rehabilitation of Manila Bay and other waterways, and issuance of local policies related to single-use plastics 26. A Sustainable Consumption and Production Action Plan **(in SDGs 8 & 13)** | B: 1.5  C: 1.5  D: 6.5; 7.3  H: 7.3  I: 9.4  N: 1.5  O: 2.4  P: 1.5  R: 1.5  S: 1.5  W: 7.3  Y: 6.3 |
| 16 | [None listed] |  |
| 17 | [None listed] |  |

Source: (Philippines, 2019) as compiled by (Elder, 2020).

Table ESM 2.7: Environment-related policies in Singapore’s VNR

| SDG | Policies which were directly mentioned | Other Applicable SDG targets |
| --- | --- | --- |
| 1 | [None listed] |  |
| 2 | 1. “Farm Transformation Map”; helped local producers to use smart technologies and innovations to optimise the use of space and improve productivity 2. A publicity & outreach programme “Love Your Food – Waste Less. Save More” 3. Food waste minimisation guidebooks for food retail and manufacturing establishments (by AVA and National Environment Agency) |  |
| 3 | [None listed] |  |
| 4 | [None listed] |  |
| 5 | [None listed] |  |
| 6 | **Efficient Water Use**   1. Funding to companies for water efficiency initiatives 2. Several programmes promote higher private sector water efficiency 3. Mandatory measures requiring efficient water use by households and industries 4. Water Efficiency Labelling Scheme. 5. NEWater and desalinated water increase water security and sustainability 6. Quick detection of contaminants, such as Fish Activity Monitoring System   **Engaging Communities on the Value of Water and Sanitation**   1. Active, Beautiful, Clean Waters (ABC Waters) Programme aims to transform utilitarian drains and canals into attractive waterways and improve runoff quality using green cleansing features 2. Friends of Water Programme 3. Public awareness programs on water conservation target different communities   **Infrastructure Planning and Innovation**   1. Smart Water Grid, a network of wireless sensors in potable water supply mains 2. Deep Tunnel Sewerage System (network of linked sewers) |  |
| 7 | 1. Power generation sector liberalization: competition has encouraged shifting from fuel oil to more efficient Combined Cycle Gas Turbine for power generation. 2. The Energy Conservation Act was enhanced to strengthen companies’ energy efficiency; planning Minimum Energy Performance Standards (MEPS) for industrial equipment 3. Government grants and support to help companies perform energy audits, enhance energy efficiency and reduce emissions 4. Singapore will implement a carbon tax across all sectors from 2019 **(in SDGs 7 & 13)** 5. Two consortiums were appointed to implement Singapore’s utility-scale Energy Storage System |  |
| 8 | [None listed] |  |
| 9 | By 2030, the public rail network will be expanded **(in SDGs 9 & 11)** |  |
| 10 | [None listed] |  |
| 11 | **Integrated Land Use Planning**   1. Urban Redevelopment Authority’s Concept Plan is for long-term land use and transport   **Transit-oriented Development and Planning**   1. The Land Transport Master Plan Land guides transport strategies and measures and is reviewed every 5 years 2. Target for 75% of morning and evening peak journeys to be made using public transport by 2030, and at least 85% by 2050. Singapore’s rail network will be expanded **(in SDGs 9 & 11)** 3. The Bus Service Enhancement Programme. From 2012-2017, 1,000 buses were added 4. “Walk Cycle Ride SG”. Walking and Cycling Plan for developments with high pedestrian and cyclist traffic was introduced. Further, developers are incentivised to provide bicycle lots and supporting facilities and more covered walkways are being constructed 5. National Cycling Plan 2010. Off-road cycling paths were constructed and bicycle parking facilities enhanced in 7 HDB towns.   **Green Buildings**   1. Green Building Masterplan 2006 to meet the 80% green buildings target   **Green Transport**   1. The Fuel Economy Labelling Scheme (information on vehicle models) 2. Rebates for low-emission vehicles and surcharges for high-emission ones 3. Electric car-sharing programme, BlueSG 2017 **(SDGs 11 & 13)** 4. Plans to deploy 50 hybrid buses by 2019, and 60 electric buses by mid-2020   **Green Spaces**   1. More neighbourhood and regional parks will be created to enable more than 90% of households to live within 400 meters or a 10 min. walk to a park by 2030. 2. 6 key areas for “City in a Garden” vision: 1. Establish world-class gardens; 2. Rejuvenate urban parks, enliven streetscapes; 3. Optimise urban spaces for greenery and recreation; 4. Enrich biodiversity; 5. Enhance competencies of landscape and horticultural industry; 6. Engage and inspire communities | A: 9.1  B: 9.1  D: 9.1  G: 9.4  K: 9.4 |
| 12 | **A Zero Waste Nation**   1. The Zero Waste Nation philosophy is integrated, e.g. Sustainable Singapore Blueprint   **Waste reduction**   1. Increase the national recycling rate from the current 61% to 70% by 2030. 2. Mandatory e-waste management framework based on Extended Producer Responsibility 3. Mandate that businesses report on the type and amount of packaging and reduction plans. Expanded government engagement with stakeholders. 4. Existing integrated waste management system. Waste is incinerated at waste-to-energy plants equipped with air pollution control equipment. 5. Food manufacturers and establishments encouraged to re-distribute unsold food 6. Reduce consumer food waste through initiatives that encourage smart and prudent food purchases, preparation, and storage habits 7. Singapore supports on-site food waste treatment at commercial premises 8. A recycling bin is provided for every public housing block and landed housing unit. Private residential developments are required to provide recycling receptacles 9. NEA collaborates with schools to set up recycling corners 10. Community 3R Outreach Programme (CROP) (awareness raising) 11. Developed various online 3R guidebooks for businesses 12. The 3R Awards for Hotels and Shopping Malls recognise outstanding 3R efforts 13. Mandated reporting waste data & waste reduction plans by large commercial premises from 2014 14. The Singapore Packaging Agreement 2007 helps to reduce packaging waste 15. Logo for Products with Reduced Packaging introduced under the SPA, eco-label 16. Singapore is investing in R&D to extract value and resources from waste streams. Environmental Services Industry Transformation Map developed to improve productivity, promote growth and create better jobs.   **Sound management of hazardous chemical and industrial waste**   1. Regulatory frameworks to ensure the management of hazardous chemicals & toxic industrial wastes, in accordance with MEAs 2. Controls on the import, export, transport, storage and use of hazardous substances, under the Environmental Protection and Management Act and the Environmental Protection and Management (Hazardous Substances) Regulations 3. Regulation on the import, export and use of products containing hazardous substances 4. Implemented the Restriction of Hazardous Substances framework (restricts the number of hazardous substances entering the environment from Electrical and Electronic Equipment). 5. Environmental Public Health (Toxic Industrial Waste) Regulations require waste collectors to be licensed 6. Approval is also required to transport toxic industrial wastes exceeding certain quantities. NEA also implements the Hazardous Waste (Control of Export, Import and Transit) Act to ensure that Singapore meets its Basel Convention obligations.   **Encouraging Energy-Efficient and Water-Efficient Behaviour and Practices**   1. Mandatory Energy Labelling Scheme 2008 2. Minimum Energy Performance Standards 2011. These are constantly reviewed 3. Mandatory Water Efficiency Labelling Scheme 2009 4. The Water Efficiency Management Plan 2010 is a voluntary initiative for commercial and industrial users to improve water use efficiency. 5. The Water Efficient Buildings (Basic) certification programme 2004 encourages building owners to increase water efficiency 6. The Water Efficiency Fund 2007 co-funds implementation of water efficiency projects 7. Public Sector Taking the Lead in Environmental Sustainability 2006 initiative requires public agencies to implement measures for energy efficiency, water efficiency and recycling. Outlined in the Public Sector Sustainability Plan 2017–2020 8. Public agencies retrofitting major energy consuming equipment encouraged to adopt the Guaranteed Energy Savings Performance contracting model   **Finding Innovative Solutions**   1. Integrated Waste Management Facility to be integrated with the Tuas Water Reclamation Plant, will allow for water-energy-waste synergies and reduce carbon emissions by more than 200,000 tonnes annually   **E-Waste Management System**   1. A mandatory e-waste management system 2021 to ensure proper recycling | R: 3.9  S: 3.9  U: 3.9  V: 3.9  W: 3.9  X: 7.3  Y: 7.3  Z: 6.4  AA: 6.4  BB: 6.4  CC: 6.4  DD: 7.3  EE: 7.2  FF: 6.5 |
| 13 | **Singapore’s Climate Change Strategy**   1. Inter-Ministerial Committee on Climate Change to enhance Whole-of-Government coordination 2. The National Climate Change Secretariat 2010 3. National Climate Change Strategy 2012, Singapore Sustainable Blueprint (updated 2015), and the two-pronged Climate Action Plan 2016 4. Climate Action Plan key mitigation strategies: (i) improving energy efficiency; (ii) reducing carbon emissions from power generation; (iii) developing cutting-edge low-carbon technologies; (iv) encouraging collective action 5. Public Sector Sustainability Plan 2017-2020: public sector electricity savings (15%), water savings (5%), and 100% green building adoption by 2020.   **Transport**   1. Vehicle Emissions Scheme provides incentives to purchase less polluting vehicles 2. Electric car-sharing programme, BlueSG 2017 **(SDGs 11 & 13)**   **Buildings**   1. Building and Construction Authority of Singapore’s Green Mark Scheme and Building Retrofit Energy Efficiency Financing   **Carbon Tax**   1. Carbon tax of S$5/tonne of CO2 equivalent emissions from 2019-2023 **(in SDGs 7 & 13)**   **Strengthening Resilience**   1. Resilience Framework. Established the Centre for Climate Research Singapore   **Promoting Climate Change Public Awareness and Action**   1. Climate change is integrated into school curricula 2. Climate Action SG Grant provides funds for local groups to organise activities that increase awareness on climate action   **Develop Options to Further Deploy Renewable Energy**   1. Housing and Development Board installs solar panels on rooftops of high-rise public housing 2. Floating photovoltaic PV project pilots floating solar panel installations at Tengeh Reservoir (Economic Development Board and PUB) 3. Renewable Energy Integration Demonstrator’s long-span wind turbine at an offshore landfill 4. SolarNova programme (EDB & HDB) aggregates solar demand of government agencies   **Further Enhance Resilience**   1. Minimum reclamation level for new projects was raised from three to four metres above mean sea level 2. Minimum crest levels were set for entrances to underground facilities 3. Terminal 5 at Changi Airport will be built 5.5m above mean sea level with upgrades to the drainage system | D: 6.4; 7.2, .3; 9.2, .4  J: 2.4; 6.6; 9.1  K: 4.7  M: 7.2  N: 7.2  O: 7.2  P: 7.2  Q: 9.1 |
| 14 | 1. Singapore currently has four legally gazetted nature reserves and 20 other administratively protected nature areas **(in SDGs 14 & 15)** 2. Integrated Urban Coastal Management strategy: optimising use of coastal resources in a sustainable way; a Whole-of-Government approach; community engagement; research, monitoring, restoration and enhancement programmes to conserve habitats 3. Sisters’ Island Marine Park 2015 is Singapore’s first marine park 4. Marine Conservation Action Plan. Species recovery is a key activity 5. Biodiversity enhancement units were installed 6. Awareness outreach programmes (Maritime and Port Authority of Singapore) 7. Maritime Singapore Green Initiative 2011 reduces shipping’s environmental impact. Includes Green Ship Programme, Green Port Programme, Green Technology Programme, Green Awareness Programme and the Green Energy Programme, and co-funding grants to develop and deploy green technologies 8. Marine Emergency Action Procedure to effectively manage spills and accidents | B: 6.6  D: 6.6  E: 6.6  G: 6.6  H: 6.6 |
| 15 | 1. Singapore currently has four legally gazetted nature reserves and 20 other administratively protected nature areas **(in SDGs 14 & 15)** 2. Singapore’s National Biodiversity Strategy and Action Plan 2009 3. Nature Conservation Master Plan strengthens biodiversity conservation efforts 4. Species recovery programme to conserve native flora and fauna 2015 5. A Habitat Enhancement and Restoration Framework in NCMP 2015 6. Strategic green corridors, also known as Nature Ways, established along roadsides 7. Eco-Link@BKE, an hourglass-shaped ecological bridge was constructed 8. Community in Nature initiative engages the community to conserve natural heritage 9. Citizen Science programme. Volunteers conduct surveys of animal groups 10. Landscaping for Urban Spaces and High-rises programme and Skyrise Greenery Incentive Scheme offer building owners and developers incentives for rooftop greenery installation. Skyrise Greenery Awards for creative designs. | C: 6.6 |
| 16 | [None listed] |  |
| 17 | 1. Annual “Ecosperity” Conferences hosted by Singapore’s Temasek Foundation with the Business and Sustainable Development Commission since 2014 2. The Monetary Authority of Singapore’s green bond grant scheme |  |

Source: (Government of Singapore, 2018) as compiled by (Elder, 2020).

Table ESM 2.8: Environment-related policies mentioned in Thailand’s VNR

| SDG | Policies which were directly mentioned | Other Applicable SDG targets |
| --- | --- | --- |
| 1 | 1. *SEP [Sufficiency Economy Philosophy] Model Villages (878)* 2. Now developing 2^nd^ National Disaster Prevention and Mitigation Plan 3. “Miss/Mister Early Warning” local people trained in early warning & disaster management |  |
| 2 | 1. Framework on the Promotion of Sustainable Agriculture 2017-2020 2. Organic Farming Improvement Project 3. New Theory Agriculture Project (capacity building for farmers & communities) 4. Master Plan on Integration of the Management of Biodiversity 2015-2021 5. Food Bank Royal Project, Mae Hong Son Province. Restoration of nature and agroforestry |  |
| 3 | [None listed] |  |
| 4 | 1. Created SEP-guided schools (21,185) to learn the importance of natural resource conservation 2. *Established Southeast Asian Ministers of Education Organization’s Regional Centre for SEP* |  |
| 5 | [None listed] |  |
| 6 | 1. National Water Vision (2000) 2. Water Resources Management Strategy 2015-2026 3. Strategy for Green Growth (under the 20-Year National Strategy Framework) 4. Strategy for Green Growth toward Sustainable development (under 12^th^ NESDP) | C: 8.4 |
| 7 | 1. Thai Integrated Energy Blueprint (20-year long term energy plan): Power Development Plan; Energy Efficiency Plan; Alternative Energy development Plan; Gas Plan; Oil Plan 2. Alternative Energy Development Plan 2015 3. Energy 4.0 Policy. Improving efficiency of the current energy system. 4. Application of SEP to support energy management in communities: Create community researchers; encourage local economy through reducing energy consumption in local companies; public awareness through “Power Conservation Community” Project; nationwide training for provincial level energy planners; multiply the impact of community energy projects 5. Energy labels |  |
| 8 | 1. Adapting Supply Chains for Drought Resilience 2. Vocational training for groups with special needs, including victims of natural disasters 3. *Sufficiency Economy Business Standard* |  |
| 9 | 1. 20-Year Draft Strategy for Transport Systems Development (2017-2036) 2. Ministry of Transport plans to procure NGV-fueled and electric public buses 3. Policy to apply Universal Design in public transport system infrastructure and vehicles 4. Development of human resources, technology, and research in utilizing innovation and effective management in the transport systems development process 5. Ministry of Transport’s Strategic Plan 2017-2021 is in line with the SDGs 6. 20-Year Thailand 4.0 Industrial Development Strategy 7. Provide entrepreneurs with education and consultation on how to increase productivity and efficiency, reduce energy and natural resources consumption 8. Programs to encourage cooperation of the local entrepreneurs and community in safeguarding the environmental and sustainable development of industry 9. The 9999 Industrial Standard balances economic, social, and environmental considerations |  |
| 10 | [None listed] |  |
| 11 | 1. The 12^th^ NESDP includes the shift towards low-carbon, smart, and inclusive city, local culture and identity preservation, environmentally friendly infrastructure, and disaster risk management 2. Department of Public Works and Town & Country Planning reformed the spatial planning system including natural resources and environment conservation. 3. Draft 20-Year Strategic Plan for Housing Development (2017-2037) 4. Integrated National Disaster Prevention and Mitigation Plan 2015 and Climate Change Management Master Plan 2015-2050 to develop prevention and preparation system | A: 9.1  D: 1.5 |
| 12 | 1. 27 government agencies jointly formed the Taskforce for SDG 12 under the Steering Committee on Natural Resources and Environment for the Implementation of SDGs 2. Sustainable Consumption and Production Roadmap 2017 – 2036 3. Shift in the public transportation systems towards energy-efficient systems 4. Product labels to promote environmentally-friendly products 5. Green Industry Policy 6. Smart Cities – Clean Energy Project and the Green Procurement Project 7. National Master Plan on Waste Management 2016 – 2021 8. The Stock Exchange of Thailand has formulated the Corporate Governance Code as a guideline to integrate the concept of sustainability into business 9. Capacity-building and building knowledge base in science and technology | E: 9.2  F: 7.2 |
| 13 | 1. Thailand’s Nationally Determined Contribution Roadmap on Mitigation 2021 – 2030 2. Climate Change Management Master Plan 2015-2050 3. “Support to the Development and Implementation of the Thai Climate Change Policy” Project provided knowledge & capacity training to local actors in pilot areas 4. National Disaster Prevention and Mitigation Plan 2015 – 2019 5. Thailand is in the process of preparing the National Adaptation Plan 6. Climate Change International Training Centre 7. Relevant agencies integrated climate change into their policies such as the Alternative Energy Development Plan 2015-2036, the Energy Efficiency Plan 2015-2036, the Power Development Plan 2015-2036, the Thailand Smart Grid Development Master Plan 2015-2036 and Environmentally Sustainable Transport System Master Plan | G: 7.2, .3; 9.1 |
| 14 | 1. Marine and Coastal Resources Management Act 2015 2. National Committee on Marine and Coastal Resources Management established 3. Provincial Committees on Marine and Coastal Resources Management 4. Clean-up activities operated in 24 coastal provinces 5. Waste reduction measures implemented in 7 pilot areas 6. “Red Tide Program” to monitor water quality in marine & coastal areas 7. Measures and programs to preserve marine ecosystems and enhance biodiversity: restoration of degraded marine and coastal ecosystems; establishment and improvement of fishing measures in line with responsible fishery; adoption of marine spatial planning as an integral part of marine and coastal zone management. 8. Up to 9 additional Marine Protected Areas to be created from 2016 – 2023 9. Under the IOCWESTPAC and GOA-ON, Thailand set up 2 permanent monitoring sites for carbonate chemistry of seawater and coral reef biodiversity 10. Command Centre for Combating Illegal Fishing 2015 11. National Plan of Action to Prevent, Deter and Eliminate IUU Fishing 2015 – 2019 12. Fisheries Management Plan 2009-2018 to tackle overfishing 13. Surveyed all existing Thai-flagged fishing vessels 14. Measures to gradually reduce the Thai fishing fleet 15. E-license scheme and fishing day scheme based on MSY 16. Entry/Exit Scheme to manage commercial fishing vessels in Thai waters 17. Port in/Port out Scheme 18. Vessel Monitoring System 19. Fishery Monitoring Centre 20. National Plan of Fishery Control and Inspection 21. Database for Fishing Vessels and Labourers 22. Fisheries Act 2015 | A: 6.6  F: 6.3  H: 6.6 |
| 15 | 1. “One Map” initiative to harmonize the scale of all maps to address the issue of overlapping concession areas 2. The 20-year National Strategy Framework contains a green growth strategy 3. National Reserved Forest Act was revised in 2016 4. Accelerated implementation of laws and regulations on forest encroachment 5. Encouraged reforestation in conservation forest and national reserved forest 6. Community forests have been established 7. Financial mechanisms for reforestation like forest bonds, tree banks, reforestation fund 8. Research & development on sustainably growing crops in forest areas 9. Total conserved area for biodiversity protection is approximately 116,800 km2 10. National plan for the conservation and sustainable use of biological diversity 11. Master Plan for Integrated Biodiversity Management 2015 – 2021 12. Since 2016, Thailand has been developing a Land Degradation Neutrality Strategy 13. National Ivory Action Plan was revised in 2014. 14. As part of NIAP, many related laws were revised, such as the Wild Animals Reservation and Protection Act 2014, and the Ivory Trade Act 2015 15. Three ivory-related registration systems were improved | H: 2.4 |
| 16 | [None listed] |  |
| 17 | National Committee for Sustainable Development to formulate polices and strategies on across economic, social and environment dimensions in a balanced and integrated manner |  |

Source: (Thailand, 2017) as compiled by (Elder, 2020).

Table ESM 2.9: Environment-related policies in Viet Nam’s VNR

| SDG | Policies which were directly mentioned | Other Applicable SDG targets |
| --- | --- | --- |
| 1 | [None listed] |  |
| 2 | 1. Development of hi-tech agriculture for clean, resilient and climate-smart agriculture 2. *NTP on New Rural Development in 2016-2020* **(in SDGs 2 & 6)** 3. Integrated Coastal Management Program |  |
| 3 | [None listed] |  |
| 4 | [None listed] |  |
| 5 | [None listed] |  |
| 6 | 1. Law on Environmental Protection 2. Law on Biodiversity 3. Law on Water Resources 4. Resolution No.24-NQ/TW of the Central Committee on pro-active climate change adaptation **(in SDGs 6 & 13)** 5. Resolution No.142/2016/QH13 on the SEDP 2016-2020 with environmental indicators 6. National Strategy on Water Resources to 2020 7. National Strategy for Environmental Protection up to 2020 with a vision to 2030 8. National Program on Safe Water Supply 2016-2025 9. National Plan of Action to improve water resource management and protection 10. *NTP on new rural development during 2016-2020* **(in SDGs 2 & 6)** 11. In addition to the Law on Water Resources 2012, the GOVN approved four decrees |  |
| 7 | 1. Electricity Plan VII. Goal of 10 percent electricity produced from renewable energy 2. Renewable Energy Development Strategy to 2030 with a Vision to 2050 3. Law on Economic and Efficient Use of Energy and Viet Nam National Energy Development until 2020 with a Vision to 2050 4. Strategy of cleaner production in the industry sector by 2020 **(in SDGs 7 & 12)** | D: 9.2 |
| 8 | 1. National Green Growth Strategy 2011-2020 and Vision to 2050 2. *Overall Plan for economic restructuring associated with the transformation of the growth model towards improved quality, efficiency, and competitiveness capacity* 3. National Action Program on Sustainable Production and Consumption by 2020 4. Tourism Development Strategy towards sustainability to 2020 with a vision to 2030 5. *Master Plan of Viet Nam Tourism Development to 2020 with a vision to 2030* 6. Resolution No. 142/QH13/2016 of the National Assembly sets out the 5-year Socio-Economic Development Plan 2016-2020 with the target of reduced energy consumption | F: 7.3 |
| 9 | 1. Viet Nam Railway Transport Development Strategy to 2020 with a vision to 2050 2. *The formation of a comprehensive infrastructure system is considered one of the three breakthroughs of the 2011-2020 Socio-Economic Development Strategy* 3. Sustainable and inclusive industrialization in the Socio-Economic Development Strategy, Viet Nam Sustainable Development Strategy, and Viet Nam Industrial Development Strategy to 2025 |  |
| 10 | [None listed] |  |
| 11 | 1. *Law on Housing* 2. Transport Development Strategy of Viet Nam to 2020 and the Project on Restructuring of the Transport Sector for Industrialization, Modernization and Sustainable Development 3. The Law on Natural Disaster Prevention and Control, particularly the Master Plan on Disaster Response, Search and Rescue to 2020 4. The National Strategy for Environmental Protection up to 2020 and Vision to 2030 5. The Orientation for the Development of Urban Drainage and Industrial Parks by 2025 6. National Strategy on Climate Change **(in SDGs 11 & 13)** 7. Scheme of Urban Development in response to Climate Change during 2013-2020 8. The goal of sustainable rural development is concretized through the NTP for New Rural Development 2010-2020 | B: 9.2  D: 1.5  E: 6.3  F: 1.5; 2.4; 7.2, .3; 9.2 |
| 12 | 1. National Strategy for Sustainable Development in 2011-2020 2. National Strategy on Green Growth **(in SDGs 12 & 13)** 3. Strategy on Cleaner Industrial Production until 2020 **(in SDGs 7 & 12)** 4. Law on Economical and Efficient Use of Energy 5. NAP on Sustainable Production and Consumption until 2020 and Vision to 2030 6. Green labels 7. Compulsory energy labels for some cars 8. Compulsory roadmap for energy labelling for motorbikes 9. Sustainable public procurement 10. Natural resources taxes of mineral mining and water 11. Environmental protection tax and environmental protection fees on wastewater 12. Law on Environmental Protection 2010 13. Law on Natural Resources 2009 14. Abolished direct subsidies on fossil fuels and phasing out indirect subsidies 15. Public awareness communication for sustainable consumption and production 16. SWITCH Asia Programme (with EU), promoting sustainable production/ consumption | A: 1.5; 3.9; 6.6; 7.2, .3; 8.4, .9; 9.1, .2, .4  D: 7.3 |
| 13 | 1. Central Committee’s Resolution 24-NQ/TW on active response to climate change **(in SDGs 6 & 13)** 2. National Strategy on Climate Change **(in SDGs 11 & 13)** 3. National Strategy on Green Growth **(in SDGs 12 & 13)** 4. Resolution 120/NQ-CP on sustainable development of the Mekong Delta in adaptation to climate change 5. NTP to Respond to Climate Change and Green Growth in 2016-2020 6. Plan for the Implementation of the Paris Agreement on Climate Change 7. Scheme to manage GHG emissions & carbon credit trading in the world market 8. Law on Natural Disaster Prevention and Control 9. Law on Irrigation 10. Law on Water Resources 11. Law on Economical and Efficient Use of Energy 12. Law on Environmental Protection 13. Some ministerial level guiding documents have harmonized contents of the national strategies and action plans 14. National Committee on Climate Change was established 15. Most ministries, agencies and provinces have specialized climate change units 16. Viet Nam developed climate change impact scenarios and updates for regions 17. Law on Environmental Protection 2005. Strategic environmental assessment tools 18. Flash flood risk zoning maps, landslide maps 19. NAP on GHG emissions mitigation 20. REDD+ | H: 1.5  J: 6.6 |
| 14 | 1. Law on Environmental Protection 2. Law on the Sea of Viet Nam 3. Law on Natural Resources and Environment of Sea and Islands 4. Law on Fisheries, tackling IUU fishing 5. The 11th Party Central Committee’s Resolution 24-NQ/TW 2013 on active response to climate change and improved management and protection of resources **(in SDGs 14 & 15)** 6. Strategy for Sustainable Exploitation and Use of Natural Resources and Protection of Marine Environment 7. National Strategy on Biodiversity 8. Viet Nam Fisheries Development Strategy until 2020 9. Planning of Viet Nam’s Marine Protected Area System up to 2020 10. Planning of Systems of Inland Water Conservation Zones up to 2020 11. Systems of marine protected areas and inland water conservation zones developed 12. Mechanisms and policies for effective and sustainable management of inland fisheries 13. Law on Fisheries 2017; issued the NAP to prevent, mitigate and eliminate IUU 14. Viet Nam has established 10 marine protected areas | F: 6.6  K: 6.6  N: 6.6 |
| 15 | 1. Law on Biodiversity 2. Law on Environment Protection 3. Law on Forest Protection and Development 4. Law on Forestry 5. Resolution 24-NQ/TW 2013 of 11th Party Central Committee **(in SDGs 14 & 15)** 6. Strategy of Environmental Protection 7. Strategy of Viet Nam Forestry Development 2006-2020 8. Strategy of Special-use Forests Management & National Strategy on Biodiversity up to 2020 with a vision to 2030 9. National Action Plan to Combat Desertification in 2006 10. Master Plan for Biodiversity Conservation up to 2020 and Orientation to 2030 11. Eight Ramsar Sites and six ASEAN Heritage Parks 12. Established 164 special use forests, including 31 national parks, 57 nature reserves, 45 landscape protected areas and 20 forests for scientific research and experiments 13. According to the National Master Plan of Special Use Forest System, the protected area system will be expanded to cover 2.4 million hectares or 176 protected areas. In addition, 16 MPAs and 45 inland water conservation areas have been included 14. Viet Nam and the EU entered a Voluntary Partnership Agreement on EU Forest Law Enforcement, Governance and Trade (VPA/FLEGT) 2017 15. Forest environmental services payment, (generates income for households participating in forest protection, improve value of forestry products) 16. National Coordination Board for implementation of the UN Convention to Combat Desertification established in 2003 |  |
| 16 | [None listed] |  |
| 17 | [None listed] |  |

Source: (Viet Nam, 2018) as compiled by (Elder, 2020).
